# Cross-protective efficacy of dendritic cells targeting conserved influenza virus antigen expressed by *Lactobacillus plantarum*

**DOI:** 10.1038/srep39665

**Published:** 2016-12-22

**Authors:** Wen-Tao Yang, Shao-Hua Shi, Gui-Lian Yang, Yan-Long Jiang, Liang Zhao, Yu Li, Chun-Feng Wang

**Affiliations:** 1 College of Animal Science and Technology, Jilin Provincial Engineering Research Center of Animal Probiotics, Engineering Research Center of Chinese Ministry of Education for Edible and Medicinal Fungi, Jilin Agricultural University, Changchun, 130118, China

## Abstract

Avian influenza virus (AIV) can infect birds and mammals, including humans, and are thus a serious threat to public health. Vaccination is vital for controlling AIV circulation. In this study, we generated a recombinant lactobacillus expressing the NP-M1-DCpep of H9N2 avian influenza virus and evaluated the activation effect of NC8-pSIP409-NP-M1-DCpep on dendritic cells (DCs) in a mouse model. The specific mucosal antibody responses and B and T cell responses in lymphoid tissues were also characterized. Importantly, we confirmed that specific CD8 T cells presented *in vitro* and antigen-specific cytotoxicity (activated the expression of CD107a) and *in vivo* antigen-specific cytotoxicity after vaccination. The adoptive transfer of NC8-pSIP409-NP-M1-DCpep-primed CD8^+^ T cells into NOD-SCID mice resulted in effective protection against mouse-adapted AIV infection. In addition, we observed protection in immunized mice challenged with mouse-adapted H9N2 AIV and H1N1 influenza virus, as evidenced by reductions in the lung virus titers, improvements in lung pathology, and weight loss and complete survival. Our data are promising for the generation of effective, non-traditional influenza vaccines against AIVs.

AIV is a major zoonotic pathogen that is transmitted by birds and represents a significant threat to mammalian health^1^,^2^,^3^,^4^. Extensive efforts have focused on the development of effective vaccines against AIV. Commercial vaccines (attenuated and inactivated vaccines) protect against AIVs by inducing the production of antibodies that intercept the viruses at the point of entry^5^. Due to antigenic changes (shift and drift) in the virus^6^, the current vaccines based on AIV surface proteins, such as hemagglutinin (HA), provide incomplete protection against infection with different subtype AIVs. This incomplete protection emphasizes the significance of developing a broad-spectrum AIV vaccine that can elicit broad heterologous protection against different subtypes of AIVs^7^.

Pathogens such as AIVs typically enter the body at mucosal surfaces, and the mucosal immune response is significant in the control of pathogenic transmission^8^. Systemically administered vaccines fail to induce adequate protective immune responses at these mucosal sites^9^. In contrast to parenteral vaccination, immunization through the mucosal immune sites could generate both a strong mucosal immune response and an effective systemic immune response^10^. However, the current AIV immunization strategies generate a principally humoral immune response that fails to elicit persistent protective effects against antigen variation in AIVs^11^. Furthermore, these protective immune effects are significant against conserved epitopes of internal proteins in AIVs, which may exhibit lower degrees of antigenic drift than antigens on surface proteins, including HA^11^,^12^. Thus, orally targeted vaccinations appear to be rational and efficient for immunization and are also one of the most promising measures available to prevent and control AIVs. A universal vaccine that offers long-lasting protection could provide heterosubtypic protection against multiple influenza subtypes^13^. The core nucleoprotein (NP) and matrix protein (M1) are attractive targets for preventive and therapeutic interventions against diverse AIVs. These proteins are internal proteins that are highly conserved among the different subtypes of AIVs and have been evaluated in many animal models^14^,^15^. In contrast to external viral glycoproteins, the amino acid sequences of these internal proteins are typically more than 90% similar^16^. In AIVs, NP and M1 are thought to contribute to the induction of subtype cross-reactive T cells against internal influenza virus antigens from diverse AIVs^11^,^12^. The use of Modified Vaccinia Ankara (MVA) to express conserved internal antigens of influenza virus can induce specific cross-reactive T cell responses to offer broad-spectrum immunity against diverse AIVs, as reported by Berthoud *et al*.^11^,^13^,^17^.

DCs are cells that continuously present antigen and are located in or beneath the epithelium in the gut. DCs contribute to the acquisition of different antigens from microorganisms through the intestinal epithelium via M cells^18^. DCs can also migrate into lymphoid tissue, where foreign antigens are presented to T and B cells to activate acquired immune responses^19^. DCs are also responsible for the polarization of naïve T cells into Th1, Th2, Th17, or regulatory T cells (Tregs) in papain-treated mice. Recently, a study has demonstrated that regulatory CD4^+^Foxp3^+^ T cells (Tregs) play important regulatory roles in the magnitude of cellular immune responses to viral infections^20^. In addition, the cellular and molecular elements of the mucosal immune system have been established^21^, and a novel mucosal vaccine utilizing a specific dendritic cell-targeting peptide (DCpep) has been developed. DCpep was found to effectively protect against a *Bacillus anthracis* lethal challenge in a mouse model^22^.

*Lactobacillus plantarum* is used as a live carrier to deliver foreign proteins on the mucosal surface to trigger effective humoral and T cell-mediated immune responses, which may be preferable in terms of safety, cost and the minimization of side effects. In previous studies, many *L. plantarum*-associated model vaccines have been recombined and tested, such as *L. plantarum* expressing the extracellular domain of invasin from *Yersinia pseudotuberculosis*^23^, tetanus toxin fragment C (TTFC)^24^, haemagglutinin from influenza virus H9N2^25^, the oxalate decarboxylase from *B. subtilis*^26^, or the AiiA lactonase from *Pseudomonas aeruginosa*^27^. Although these studies demonstrate that the vaccines have generally achieve their goals, not all were effective.

The *Escherichia coli-Lactobacillus plantarum* shuttle and expression vector (pSIP-409) constructed by Sørvig and colleagues is a stable, mature inducible expression system^28^. We and other researchers have demonstrated that recombinant *L. plantarum* (NC8) can induce effective immune responses against pathogen infection in different animal models^25^,^27^. In the present study, to evaluate the effects of DCpep in enhancing a broadly heterologous protective immune response, an oral vaccine was developed by using *L. plantarum* to deliver the internal AIV proteins (NP and M1) fused to DCpep to mucosal DCs.

## Results

### Expression of rNP-M1 in *L. plantarum* NC8

**Figure 1:**
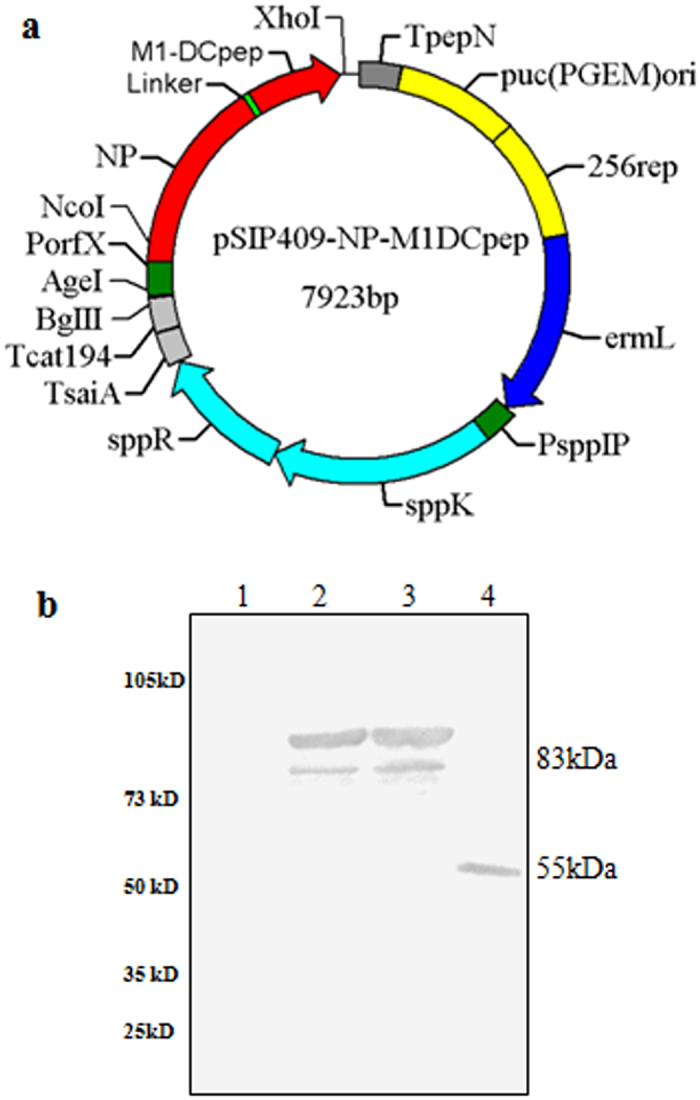
Plasmid construction and protein synthesis. (**a**) The pSIP409-NP-M1-DCpep was generated as described in the Methods. NP: nucleo-protein gene; M1: matrix protein gene; DCpep: dendritic cell-targeting peptide; ermL: erythromycin-resistance marker; 256rep: replication origin for *Lactobacillus*; PorfX and PsppIP: inducible promoters; sppR and sppK: response regulator and histidine protein kinase, respectively. (**b**) Western blotting of the synthesized proteins. All cultures were induced with SppIP for 8 h and then subjected to an immunological assay using an anti-NP prime antibody. Lane 1: negative control, NC8(pSIP-409); lane 2: NC8(pSIP409-NP-M1-Ctrlpep), lane 3: NC8(pSIP409-NP-M1-DCpep); and lane 4: Purified NP protein.

To determine whether targeting the AIV antigens to DCs would induce cellular immune responses, we generated a recombinant *L. plantarum* vector expressing the complete NP and M1 from influenza A/duck/Xuzhou/07/2003(H9N2) virus fused to DCpep at the C terminus (pSIP409-NP-M1-DCpep) through a 13-amino-acid linker (Fig. 1). A recombinant *L. plantarum* vector expressing a non-targeted NP-M1-Ctrlpep fusion (pSIP409-NP-M1-Ctrlpep) and an “empty” vector control (pSIP409) were also generated. The recombinant plasmids were successfully constructed and used to transform *L. plantarum* (Fig. 1a). The expression of NP-M1-DCpep and NP-M1-Ctrlpep was observed *in vitro*. Cell pellets of cultures of NC8-pSIP409-NP-M1-DCpep, NC8-pSIP409-NP-M1-Ctrlpep, and NC8-pSIP409 (empty vector) were subjected to western blotting detection using an anti-NP antibody. As shown in Fig. 1b, the proteins encoded by NC8-pSIP409-NP-M1-DCpep and NC8-pSIP409-NP-M1-Ctrlpep formed a high-molecular-weight protein with a size of 83 kDa. The fusion antigen was also confirmed by anti-M1 Abs binding (Fig. S1).

### Activation of DC costimulatory molecules by *L. plantarum* expressing NP-M1-DCpep

**Figure 2:**
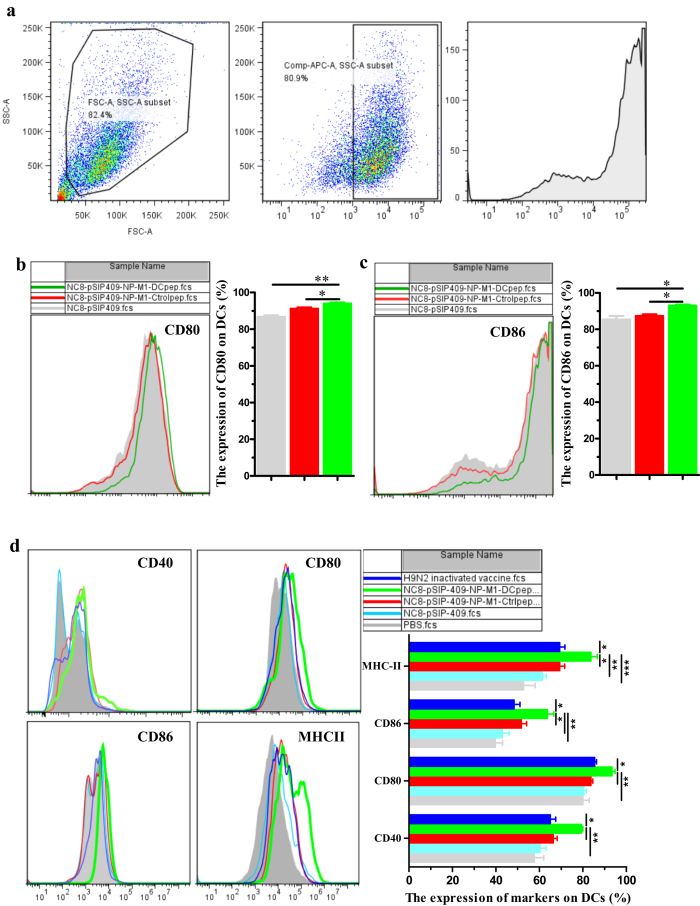
Upregulated expression of CD80, CD86, CD40 and MHC-II on the surface of DCs. (**a**) The DC gating strategy. Mouse bone marrow DCs were co-cultured with recombinant *L. plantarum* for 12 h, and the expression levels of CD80 (**b**) and CD86 (**c**) were analysed by flow cytometry. (**d**) DCs were also isolated from the small intestines of mice immunized with recombinant *L. plantarum* or H9N2 inactivated vaccine for 24 h, and the expression levels of CD80, CD86, CD40 and MHC-II were determined. The results are presented as the means ± S.E.M of triplicate tests (n = 8–10 mice in each group), and were analysed by using a one-way ANOVA, assuming a Gaussian distribution, followed by the Bonferroni Post-test, and the statistical significance of differences between groups were analysed (*P < 0.05, **P < 0.01, and ***P < 0.001).

Regulating the activation status of DCs improves DC function^29^. To evaluate the potential effect of NC8-pSIP409-NP-M1-DCpep on DCs, mouse DCs were generated from bone marrow cells, and the activation of mouse DCs invitro was evaluated using a gating strategy, as shown Fig. 2a. The co-culture of NC8-pSIP409-NP-M1-DCpep with mouse DCs elicited a significant enhancement in the expression of the markers CD80^+^ and CD86^+^ on mouse DCs (Fig. 2b,c). To induce full T cell activation, a second signal from co-stimulatory/regulatory molecules for DCs is important^30^.

The mice were then orally inoculated once with *L. plantarum* expressing NP-M1-DCpep under anaesthesia 24 h after NC8-pSIP409-NP-M1-DCpep inoculation. Their PPs, mesenteric lymph nodes (MLNs) and intestines were collected, and the cells were labelled with CD11c, CD40, CD80, CD86 and MHC-II to analyse the expression of these molecules and determine the frequency of these activation markers on DCs. Compared with the controls, NC8-pSIP409-NP-M1-DCpep induced a notable enhancement in the surface expression of CD80^+^, CD86^+^, CD40^+^, and MHC-II^+^ on CD11c^+^ cells in the lamina propria of the small intestine by 24 h post-vaccination (Fig. 2d). However, in contrast to the MLN, only CD11c^+^CD40^+^ and CD11c^+^CD80^+^ DCs were present in the PPs at 24 h post-vaccination (Fig. S2b,c). Furthermore, at 36 h post-vaccination, greater frequencies of CD11c^+^CD80^+^, CD11c^+^CD86^+^, CD11c^+^CD40^+^, and CD11c^+^MHC-II^+^ DCs were observed in the MLNs of mice treated with NC8-pSIP409-NP-M1-DCpep (data not shown). These data also imply that DC activation initially occurs in the small intestine and PPs following oral NC8-pSIP409-NP-M1-DCpep administration and that these activated DCs subsequently migrate to the MLN. The increased expression of DC activation markers may indicate the functional maturation of these APCs.

### Specific antibody titers against the NP and M1 antigens

**Figure 3:**
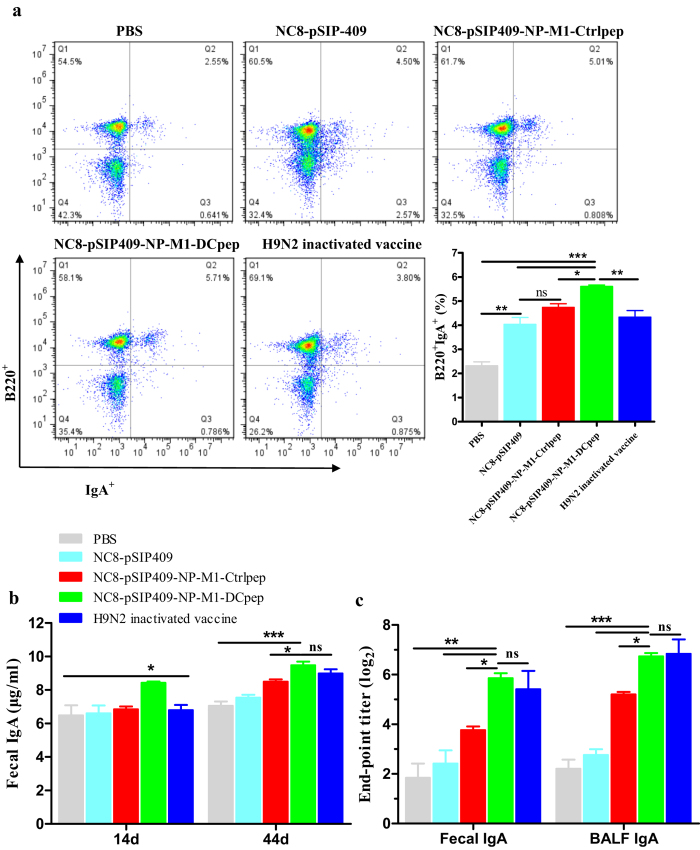
Local immune responses induced by recombinant vaccines. C57BL/6 mice were orally administered NC8 strains harbouring each plasmid as indicated on days 1, 2 and 3, and were then boosted on days 28, 29 and 30. (**a**) Fourteen days after booster immunization, the number of B220^+^ IgA^+^ cells in PP was measured by flow cytometry (n = 5/group). (**b**) The total sIgA in faeces was measured by ELISA (n = 8~10/group) on days 14 and 44 after primer immunization. (**c**) The specific sIgA titres in the faecces and BALF were analysed 44 days after the primer vaccination (n = 5/group). The results are presented as the means ± S.E.M and were analysed by using a one-way ANOVA, assuming a Gaussian distribution followed by Dunnett’s post-test and are expressed relative to the values for PBS, NC8-pSIP409, NC8-pSIP409-NP-M1-Ctrlpep and to the H9N2 inactivated vaccine (*P < 0.05, **P < 0.01, and ***P < 0.001). The data shown represent one of the three experiments with equivalent results.

**Figure 4:**
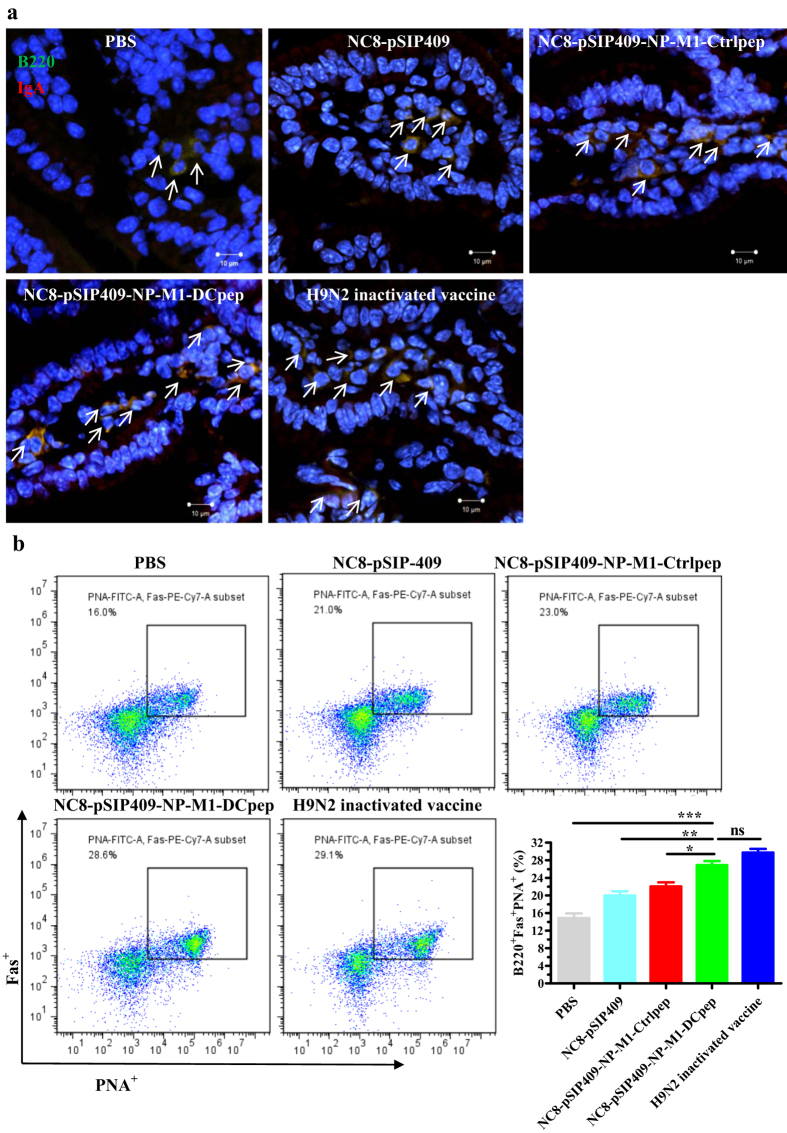
Determination of IgA-expressing cells within the small intestine and activated B cells in the growth centre (GC) of vaccinated mice. (**a**) Ileum sections were also stained with anti-IgA (red) and B220 (green) antibodies on 44 day after primer immunization and were analysed by confocal microscopy (Zeiss LSM710). Scale bar, 10 μm (400×). (**b**) Detection of the activated B cells in the GC of PPs. The GC B cells among B220^+^ B cells (PNA^+^ FAS^+^) in the PPs were determined by flow cytometry on day 5 after the booster immunization. The results are presented as the means ± S.E.M and were analysed by using a one-way ANOVA, assuming a Gaussian distribution followed by Dunnett’s post-test and are expressed relative to the values for PBS, NC8-pSIP409, NC8-pSIP409-NP-M1-Ctrlpep and to the H9N2 inactivated vaccine (*P < 0.05, **P < 0.01, and ***P < 0.001). The data shown represent one of the three experiments with equivalent results.

The mice were orally immunized with *L. plantarum* expressing NP-M1-DCpep three times as the primer vaccination, and four weeks after the primer vaccination, the mice received three booster immunizations. Fourteen days after the last booster immunization, we observed significantly increased numbers of IgA^+^B220^+^ B cells in the PPs of the NC8-pSIP409-NP-M1-DCpep group compared with all of the other groups (Fig. 3a). In addition, the antibody content was also detected 14 and 44 days after the primer vaccination. Total IgA responses in the excrement were induced in all of the groups and detected by ELISA. Compared with the other groups, a significant increase in the IgA antibody levels was observed in the excrement of the NC8-pSIP409-NP-M1-DCpep group (Fig. 3b). Furthermore, higher NP-M1-specific IgA titers were observed in the faeces and bronchoalveolar lavage fluid (BALF) of the mice that received NC8-pSIP409-NP-M1-DCpep or H9N2 inactivated vaccine compared with the NC8-pSIP409-NP-M1-Ctrlpep, NC8-pSIP409 and PBS groups (Fig. 3c). The increase in ileal IgA detected in mice vaccinated with recombinant vaccines correlated with the induction of mucosal immune responses (Fig. 4a).

The Ab response might be related to the germinal centers (GCs) in the LNs and spleens of the mice immunized with recombinant bacteria. PPs and MLNs were collected from each mouse five days post-primer vaccination. The MLN and PP cells from the immunized and control groups of mice were gated on B220^+^ FAS^+^ PNA^+^ B cells. The proportions of FAS^+^ PNA^+^ B220^+^ B cells in the PPs (Fig. 4b) and MLNs (Fig. S3) were significantly higher in all of the groups of immunized mice compared with the PBS control group. In addition, the proportions of FAS^+^PNA^+^B220^+^ B cells in the draining lymph nodes (dLNs) and spleens were significantly higher in the groups that received recombinant bacteria or the inactivated H9N2 vaccine than in the PBS group (data not shown). These data demonstrate that GCs developed in lymph nodes after oral vaccination with *L. plantarum*.

### NC8-pSIP409-NP-M1-DCpep immunization induces antigen-specific T cell responses

**Figure 5:**
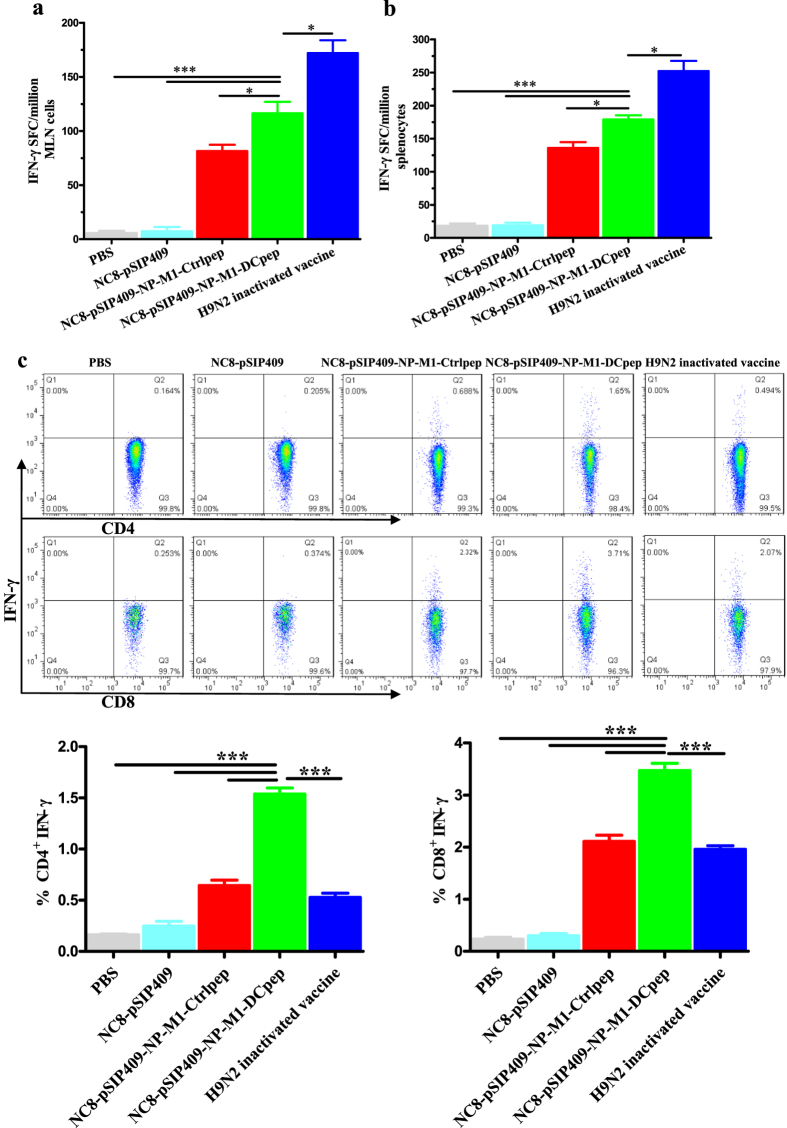
Oral vaccination with NC8-pSIP409-NP-M1-DCpep elicits enhanced T cell immune responses. The antigen-specific cytokine secretion from MLNs and splenocytes of C57BL/6 mice was detected two weeks after oral immunization. (**a**,**b**) The number of IFN-γ spot forming cells (SFC) from MLNs and splenocytes was determined by using an Elispot assay. (**c**) The frequency of antigen-specific IFN-γ-producing CD4^+^ T cells and CD8^+^ T cells in the MLN was detected by flow cytometry. The data are the mean values ± SEM (n = 5) and were analysed by using a one-way ANOVA, assuming a Gaussian distribution, followed by Dunnett’s post-test and are expressed relative to PBS, NC8-pSIP409 and NC8-pSIP409-NP-M1-Ctrlpep and to the H9N2 inactivated vaccine (*P < 0.05, and ***P < 0.001). The data shown represent one of the three experiments with equivalent results.

To evaluate the cell-mediated immune responses elicited by oral immunization with NC8-pSIP409-NP-M1-DCpep and the controls, the specific interferon-γ enzyme-linked immunospot (IFN-γ ELISPOT) responses to the specific NP and M1 epitopes of AIVs in the spleens and MLNs were analysed two weeks after the booster immunization. The mice vaccinated with NC8-pSIP409-NP-M1-DCpep exhibited a significantly higher proportion of IFN-γ-producing cells in the MLNs and spleens in response to stimulation with the specific epitopes of NP and M1 compared with all of the other groups with the exception of the inactivated H9N2 vaccine group (Fig. 5a,b).

The CD4^+^ and CD8^+^ T cell responses in the spleens and MLNs were also evaluated by ICS. We observed higher proportions of specific antigen-reactive CD4^+^IFN-γ^+^ and CD8^+^IFN-γ^+^ T cells in the MLNs after oral immunization with NC8-pSIP409-NP-M1-DCpep compared with the control groups (Fig. 5c). In addition, the frequencies of CD4^+^ IFN-γ^+^ and CD8^+^ IFN-γ^+^ T cells in the spleen were significantly higher in the NC8-pSIP409-NP-M1-DCpep group than in the other vaccine groups (Fig. S4).

**Figure 6:**
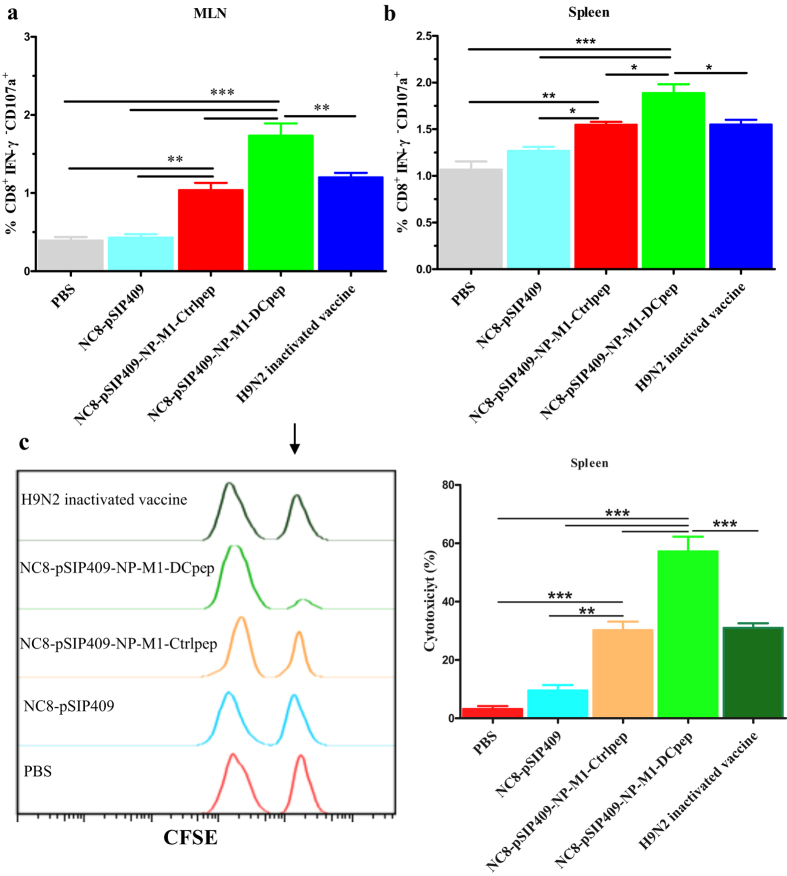
Spleen resident CD8 T cells express perforin and lyse peptide-pulsed targets. (**a**,**b**) The frequency of CD107a-producing CD8^+^ T cells from MLNs and splenocytes was analysed two weeks after boost vaccination. (**c**) The splenocytes from naive mice stained with CFSE were injected into vaccinated mice after being coated with the NP and M1 peptides. The *in vivo* cell-mediated cytotoxicity was characterized at 18 h. The black arrow shows the NP and M1-pulsed, CFSE^High^ target cells. The data are the mean values ± SEM (n = 5) and were analysed by using a one-way ANOVA, assuming a Gaussian distribution, followed by Dunnett’s post-test and are expressed relative to PBS, NC8-pSIP409 and NC8-pSIP409-NP-M1-Ctrlpep and to the H9N2 inactivated vaccine (*P < 0.05, **P < 0.01, and ***P < 0.001). The data shown represent one of the three experiments with equivalent results.

Similarly, the proportion of CD-107a^+^IFN-γ^−^ CD3^+^CD8^+^ T cells in the MLNs and spleens was significantly higher in the group orally immunized with NC8-pSIP409-NP-M1-DCpep (Fig. 6a,b). To further assess the regulation of effectors of cytotoxic activity by vaccination-induced NP- and M1-specific CD8^+^ T cells, an *in vivo* CTL assay was performed (Fig. S5). The mice inoculated with NC8-pSIP409-NP-M1-DCpep exhibited higher cytotoxic function than the orally immunized mice in the other groups (Fig. 6c). Hence, NC8-pSIP409-NP-M1-DCpep immunization effectively induced AIV-specific CTL cytotoxicity.

**Figure 7:**
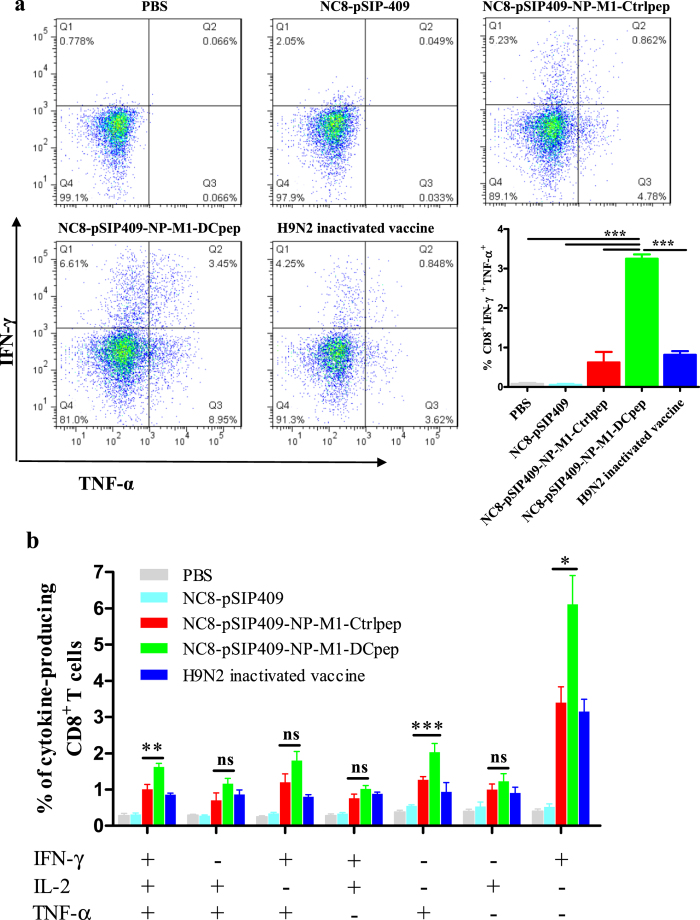
Ag-specific CD8^+^ T cell cytokine responses after NC8-pSIP409-NP-M1-DCpep vaccination. (**a**) Pulmonary CD3^+^CD8^+^ T cells from vaccinated mice were assessed by using intracellular cytokine staining for IFN-γ and TNF-α. (**b**) The frequency of each possible cell population of IFN-γ, TNF-α and IL-2 was evaluated. The data are the mean values ± SEM (n = 5) and were analysed by using a one-way ANOVA, assuming a Gaussian distribution, followed by Dunnett’s post-test and are expressed relative to PBS, NC8-pSIP409 and NC8-pSIP409-NP-M1-Ctrlpep and to the H9N2 inactivated vaccine (**P < 0.01, and ***P < 0.001). The data shown represent one of the three experiments with equivalent results.

IL-2, IFN-γ and TNF-α are regularly used to determine antigen-specific polyfunctional T cells. The induction of these multifunctional T cells by vaccination may be related to protection against *Leishmania major* or influenza virus challenge^16^,^31^. We therefore also performed ICS to analyse the proportions of the specific cytokine-producing CD8^+^ T cells observed after boosting mice with NC8-pSIP409-NP-M1-DCpep via the oral route (Fig. 7).

Higher proportions of peptide-specific IFN-γ/TNF-α double-positive cells were detected in the lungs of mice immunized with NC8-pSIP409-NP-M1-DCpep (Fig. 7a). Furthermore, in the spleen, the proportion of peptide-specific T cells secreting any of these cytokines (IFN-γ or TNF-α) was higher in the NC8-pSIP409-NP-M1-DCpep-immunized mice than in the mice belonging to the other groups of mice (Fig. 7b). In addition, higher proportions of IFN-γ/TNF-α/IL-2 triple-positive cells (Fig. 7b) were detected in the spleens of mice immunized with NC8-pSIP409-NP-M1-DCpep, demonstrating that NC8-pSIP409-NP-M1-DCpep can improve the quality of CD8^+^ T cell responses.

**Figure 8:**
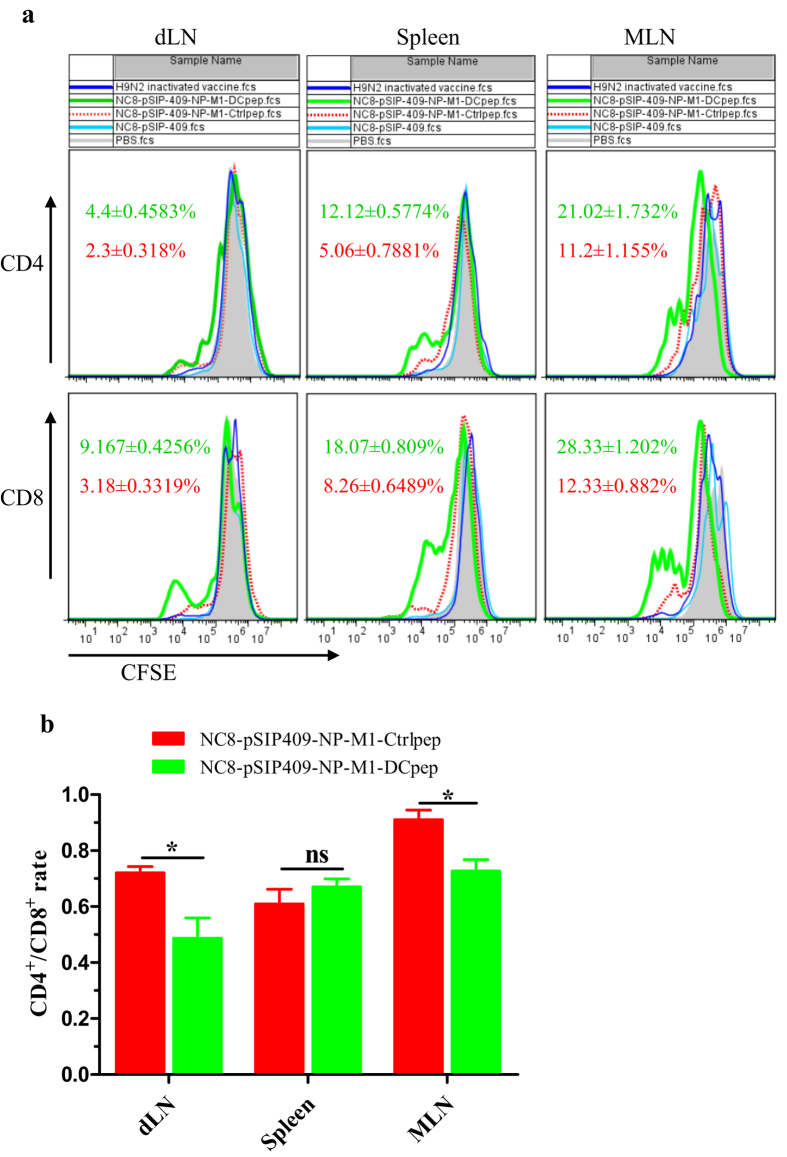
Vaccination with recombinant *L.plantarum* strains contributes to the proliferation of antigen-specific CD4 T cells and CD8 T cells in the dLN, spleens and MLN. (**a**) Thirty days after booster vaccination, the dLN, spleens and MLN were collected from each group and stained with CFSE. A total of 5 × 10^5^ cells were incubated with the NP and M1 peptides for four days, and flow cytometry was performed to detect specific proliferation. (**b**) The histogram indicates the CD4:CD8 ratio in the dLN, spleens and MLN of the NC8-pSIP409-NP-M1-Ctrlpep group and the NC8-pSIP409-NP-M1-DCpep group. The results are presented as the mean ± SEM of triplicate tests (n = 5 mice per group) and were analysed by using an unpaired t-test. The data are expressed relative to NC8-pSIP409-NP-M1-Ctrlpep (*P < 0.05). The data shown represent one of three experiments with equivalent results.

To evaluate the T cell proliferation response to NP and M1 peptide restimulation, the T cells were analysed following oral immunization with recombinant *L. plantarum*. Splenocytes, MLNs and dLNs isolated from the different groups of mice one month after the final vaccination were incubated with NP and M1 peptides *in vitro* (Fig. 8). Significant CD4^+^ T cell and CD8^+^ T cell proliferation was observed in response to NP and M1 peptide induction in the spleen, MLNs and dLNs isolated from the mice vaccinated with NC8-pSIP409-NP-M1-DCpep compared with those isolated from the mice vaccinated with NC8-pSIP409-NP-M1-Ctrlpep, other forms of recombinant *L. plantarum* or the inactivated H9N2 vaccine (Fig. 8a). In addition, the data indicated that the ratio of CD4^+^/CD8^+^ cells was not noticeably different in the spleens of the NC8-pSIP409-NP-M1-Ctrlpep and NC8-pSIP409-NP-M1-DCpep groups. However, the ratio of the dLN to the MLN was markedly reduced in the NC8-pSIP409-NP-M1-DCpep group compared with the control group (Fig. 8b). Furthermore, IFN-γ cytokine responses were observed in response to NP and M1 peptide restimulation in the mice vaccinated with NC8-pSIP409-NP-M1-DCpep but not in those belonging to the other groups (data not shown).

### Adoptively transferred immune CD8^+^ T cells can protect mice against AIV

**Figure 9:**
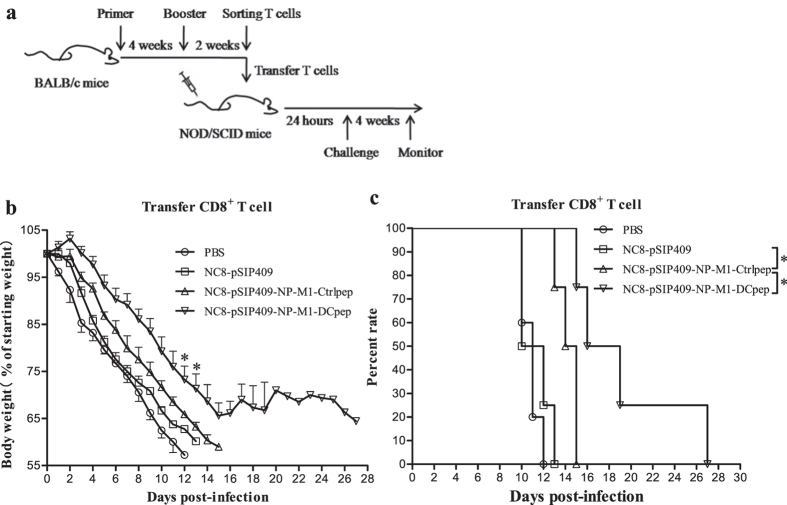
Protective effect of transferred NC8-pSIP409NP-DCpep-primed CD8^+^ T cells in NOD/SCID mice. (**a**) CD8^+^ T cells were isolated from the spleens and MLN of mice immunized with the different strains (as indicated) 14 days after booster vaccination and were then adoptively transferred to NOD/SCID mice by i.v. (tail vein) injection. After 24 h, the NOD/SCID mice were infected with 0.5 × LD_50_ of mouse-adapted H9N2 AIVs and subsequently monitored daily for four weeks after infection. (**b**) The weight loss of the mice infected with mouse-adapted H9N2 AIVs was measured daily until day 28. (**c**) The survival rates (percentages) of the NOD/SCID mice were monitored for four weeks after infection. The results are presented as the means ± SEM of triplicate tests (n = 5 mice per group). The weight loss data were analysed by using a one-way ANOVA, assuming a Gaussian distribution, followed by Dunnett’s post-test. The mortality data were analysed by using the Log-rank (Mantel-Cox) test and are expressed relative to the NC8-pSIP409 and NC8-pSIP409-NP-M1-Ctrlpep groups (*P < 0.05). The data shown represent one of the three experiments with equivalent results.

To determine whether the protective effect of transferred NC8-pSIP409-NP-M1-DCpep-primed CD8^+^ T cells against mouse-adapted H9N2 (mH9N2) AIVs in NOD-SCID mice is associated with an enhanced specific CD8^+^ T cell response, CD8^+^ T cells were isolated at day 14 post-vaccination from the spleens and MLNs of mice vaccinated with NC8-pSIP409-NP-M1-DCpep, NC8-pSIP409-NP-M1-Ctrlpep, NC8-pSIP409 or PBS and adoptively transferred to NOD-SCID mice by i.v. (tail vein) injection (Fig. 9). Significantly lower weight loss was observed in the mice that received the NC8-pSIP409-NP-M1-DCpep-primed CD8^+^ T cells from day 12 to day 13 (P = 0.0453 and P = 0.0495) compared with the control NOD-SCID mice that received NC8-pSIP409-NP-M1-Ctrlpep-primed CD8^+^ T cells (Fig. 9b). Importantly, a significantly longer survival time was obtained for the NOD-SCID mice adoptively transferred with NC8-pSIP409-NP-M1-DCpep-primed CD8^+^ T cells compared with that found for the control NOD-SCID mice (P = 0.0285) (Fig. 9c). To confirm that the protection was provided only by the adoptively transferred CD8^+^ T cells and not by CD4^+^ T cells from the NC8-pSIP409-NP-M1-DCpep-primed mice, CD4^+^ T cells from the vaccinated mice were also adoptively transferred into NOD-SCID mice. No significant differences were observed between the groups after viral infection, but the NOD-SCID mice that received NC8-pSIP409-NP-M1-DCpep-primed CD4^+^ T cells exhibited an extended survival time compared with that of the control NOD-SCID mice (Fig. S6a,b). Furthermore, the co-transfer of NC8-pSIP409-NP-M1-DCpep-primed CD4^+^ and CD8^+^ T cells failed to efficiently protect NOD-SCID mice against AIV (Fig. S6c,d). These results indicate that NC8-pSIP409-NP-M1-DCpep-primed CD8^+^ T cells protect against mH9N2 AIV challenge.

### Recombinant *L. plantarum* elicits protective immunity against infection with homologous and heterologous influenza viruses

**Figure 10:**
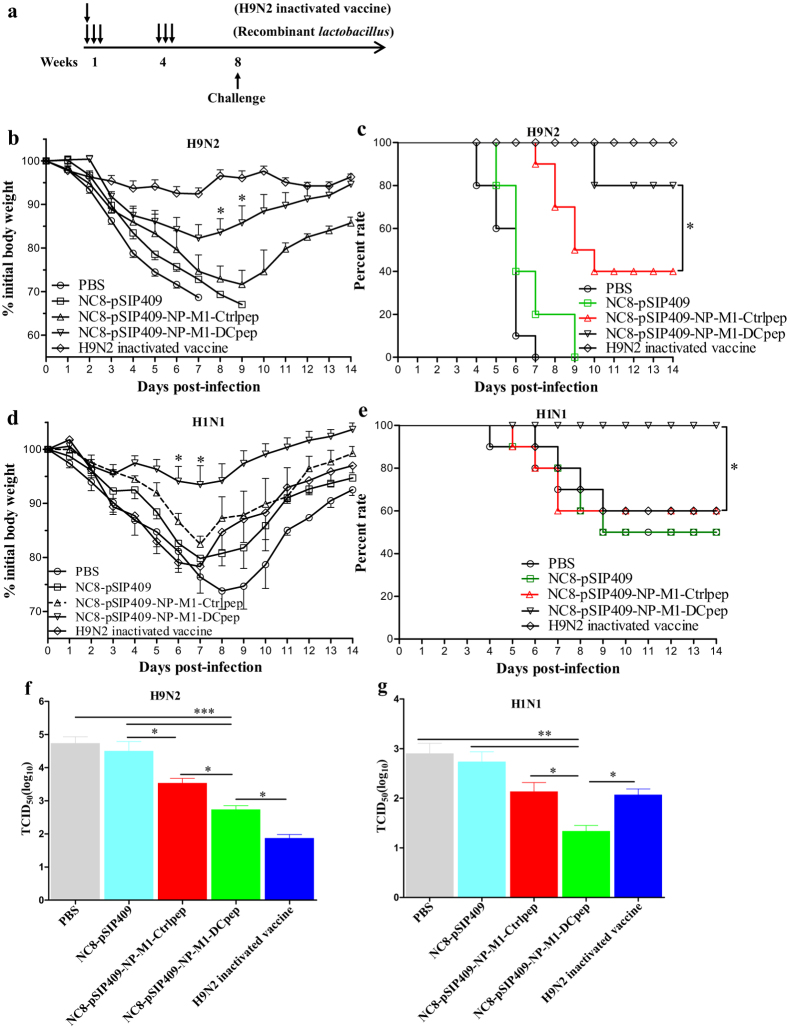
Oral vaccination with recombinant *L. plantarum* protects mice from homologous and heterologous influenza virus infections. Groups of C57BL/6 mice were immunized and challenged as indicated in (**a**). Four weeks after vaccination, the mice were infected with either mouse-adapted H9N2 AIVs (10 × LD_50_) or A/PR/8/34(H1N1) (0.5 × LD_50_), and their weight loss (**b**,**d**) and survival (**c**,**e**) were recorded for 14 days. The weight loss and mortality data are presented as the means ± SEM of triplicate tests (n = 10 mice per group). The weight loss data were analysed by using an unpaired t-test. The mortality data were analysed by using a Log-rank (Mantel-Cox) test and are expressed relative to the NC8-pSIP409-NP-M1-Ctrlpep group (*P < 0.05). Five days after infection, the mouse-adapted H9N2 AIVs (**f**) and A/PR/8/34(H1N1) (**g**) in the lungs were titrated in MDCK cells. The results are presented as the means ± SEM of triplicate tests (n = 5 mice per group) and were analysed by using a one-way ANOVA, assuming a Gaussian distribution, followed by Dunnett’s post-test and are expressed relative to the PBS, NC8-pSIP409 and NC8-pSIP409-NP-M1-Ctrlpep and the H9N2 inactivated vaccine groups, (*P < 0.05, **P < 0.01, and ***P < 0.001). The data shown represent one of three experiments with equivalent results.

**Figure 11:**
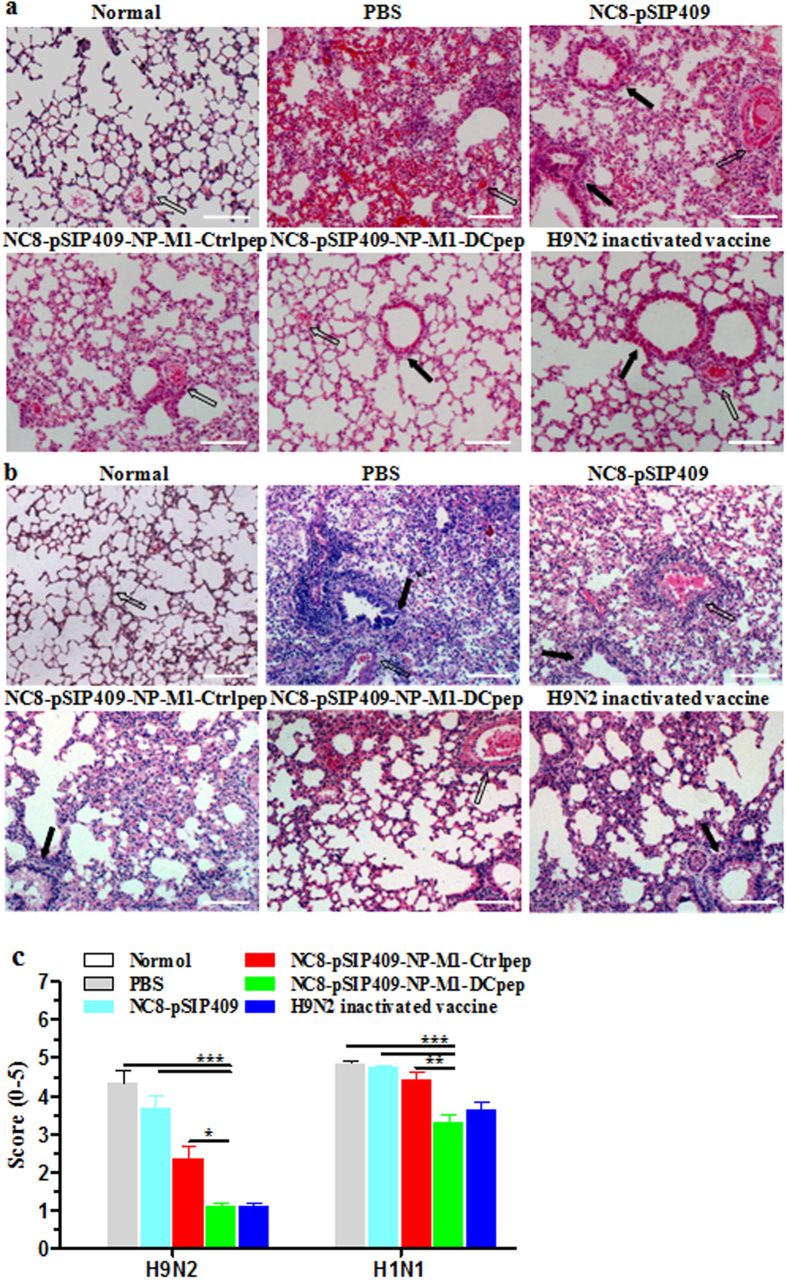
Protective effects of the recombinant *L. plantarum* on the appearance of the pathological damage in the lungs of virus-infected mice. Lung samples from the groups of vaccinated mice were fixed with 4% paraformaldehyde on day 5 post-infection with mouse-adapted H9N2 AIVs (10 × LD_50_) (**a**) and A/PR/8/34(H1N1) (0.5 × LD_50_) (**b**). The sections were then stained with H&E (100×), and the figures show the results of a histopathological analysis from of at least three individual experiments. Open arrows show a vessel, and closed straight arrows show a bronchus. Scale bar, 200 μm. (**c**) The histopathological scores for lung sections from H9N2 or H1N1-infected mice. Lung sections were scored by an investigator blinded to the mouse identity for total lung inflammation (alveolitis and peribronchiolar inflammation) on a scale from 0 to 5. The results are presented as the means ± SEM of triplicate tests (n = 3 ~ 5 mice per group), and the significance of differences was analysed using a one-way ANOVA, assuming Gaussian distribution, followed by Dunnett’s post-test and are expressed relative to PBS, NC8-pSIP409 and NC8-pSIP409-NP-M1-Ctrlpep (*P  <  0.05, **P < 0.01, and ***P < 0.001).

After homologous mouse-adapted H9N2 AIV infection (Fig. 10a), significant differences in weight loss were observed between the NC8-pSIP409-NP-M1-DCpep and NC8-pSIP409-NP-M1-Ctrlpep groups (unpaired t-test of peak weight loss, observed on day 8–9, P = 0.0325), and the difference in weight loss between the NC8-pSIP409-NP-M1-DCpep group and the H9N2 inactivated vaccine groups was significant (P = 0.017) (Fig. 10b). In addition, as shown in Fig. 10c, the NC8-pSIP409-NP-M1-DCpep-immunized group exhibited 40% and 80% survival after challenged with a lethal dose of mH9N2 AIV compared with the NC8-pSIP409-NP-M1-Ctrlpep group [log-rank (Mantel-Cox) test, P = 0.0389] and NC8-pSIP409 control group [log-rank (Mantel-Cox) test, P = 0.0001], respectively. In contrast, the difference in survival between the NC8-pSIP409-NP-M1-DCpep group and the H9N2 inactivated vaccine control group was not significant (P = 0.2994). Importantly, the viral load in the lungs after infection was 2 logs and 1 log lower in the mice immunized with NC8-pSIP409-NP-M1-DCpep compared with the loads observed in the mice vaccinated with NC8-pSIP409 (P < 0.0001) and those vaccinated with NC8-pSIP409-NP-M1-Ctrlpep (P < 0.05), respectively (Fig. 10f). Consistent with these findings, the degree of lung damage after challenge with AIV was reduced in the mice immunized with NC8-pSIP409-NP-M1-DCpep compared with that observed in the mice immunized with NC8-pSIP409-NP-M1-Ctrlpep, NC8-pSIP409 or PBS (Fig. 11a,c).

To determine whether this type of influenza vaccine-induced immune response can mediate protection during heterosubtypic influenza virus infection, groups of mice were orally vaccinated with NC8-pSIP409-NP-M1-DCpep and challenged 14 days later with heterosubtypic strains of A/PR/8/34(H1N1) (10 × LD_50_). In a repeated tests utilizing a lethal dose of H1N1 virus, we observed no significant difference in weight loss after mice were challenged with H1N1 (10 × LD_50_) (Fig. S7a). Both vaccinated and control mice succumbed to infection, but the survival time of the mice vaccinated with NC8-pSIP409-NP-M1-DCpep was always more longer than that of the mice that received PBS, NC8-pSIP409, or NC8-pSIP409-NP-M1-Ctrlpep [NC8-pSIP409-NP-M1-DCpep compared with NC8-pSIP409-NP-M1-Ctrlpep, log-rank (Mantel-Cox) test, P = 0.1716] (Fig. S7b,c).

The above-described results were further validated by challenge with a smaller dose of H1N1 (0.5 × LD_50_) after vaccination. As expected, 50% of the control mice survived the infection, but the mice administered NC8-pSIP409-NP-M1-DCpep 15 days prior to challenge presented significant improvements in terms of bodyweight loss (NC8-pSIP409-NP-M1-DCpep vs NC8-pSIP409-NP-M1-Ctrlpep, unpaired t-test of peak weight loss, observed on day 6–7; P = 0.0205) and survival [NC8-pSIP409-NP-M1-DCpep vs NC8-pSIP409-NP-M1-Ctrlpep, log-rank (Mantel-Cox) test, P = 0.0419] (Fig. 10d,e). In contrast to the control mice, the mice immunized with NC8-pSIP409-NP-M1-DCpep exhibited significantly lower residual lung virus titers after infection with H1N1 (Fig. 10g). Furthermore, the degree of lung damage after challenge with H1N1 was significantly reduced in the mice immunized with NC8-pSIP409-NP-M1-DCpep compared with those belonging to the other groups (Fig. 11b). After the lung sections were scored by three independent blinded readers, the lung inflammation was found to be significantly decreased in the lungs from infected mice vaccinated with NC8-pSIP409-NP-M1-Dpep compared with the PBS- or NC8-pSIP409-NP-M1-Ctrlpep**-**vaccinated controls (Fig. 11c). These results directly indicate that oral immunization with *L. plantarum* expressing NP-M1-DCpep offers effective protection against heterologous influenza virus infection.

## Discussion

AIVs have caused serious economic losses in the poultry industry worldwide. In addition, the high incidence and mortality of AIVs also threaten mammalian and human life^2^. Waterfowl are generally considered the natural reservoir of AIVs and can easily transmit viruses to other species, such as poultry, via migration. Transmission to other species likely requires pivotal genetic and antigenic mutations^32^,^33^. Specific T cell responses are elicited by the relatively well conserved internal influenza antigens, which are an important component of the inherent immunity to influenza, particularly heterosubtypic AIVs. However, the current AIV vaccines do not provide cross-protection against different subtypes of AIVs. Hence, the development of safe and efficacious oral vaccines against a broader spectrum of AIVs that infect poultry has been actively pursued. These efforts are not intended to completely control the virus but rather to alleviate clinical symptoms, reduce virus shedding and accelerate recovery^16^. The T cell-mediated immune response could also offer much broader protection when combined with a humoral response, which can play a critical role incomplete protection against the influenza subtypes included within the vaccine and, importantly, partial protection against other subtypes to prevent a pandemic^17^.

Studies conducted by our laboratory and others indicate that the lactobacillus expression system is a mature and effective vector for expressing AIV antigen and allows induction of a stronger immune response against pathogen infection^34^. In addition, lactobacillus can induce the activation of DCs in MLNs and the small intestine cells help kill the pathogen, in a mouse model^29^. In this study, we observed that recombinant *L. plantarum* can enhance the expression of DC markers *in vivo* and *in vitro*. Importantly, the oral vaccination of mice with *L. plantarum* expressing NP-M1-DCpep also effectively elicited the activation of DCs in the small intestine and gradually in the PPs and MLNs. Similar results have been obtained by Kathania^30^. These data confirm that DC activation can effectively promote T cell and B cell differentiation and potentially contribute to rapid pathogen clearing in the host.

Mucosal vaccination can induce stronger mucosal immune responses^35^, which are of great significance for AIVs. In addition, sIgA antibodies are the key effectors in the protection of the mucosa by the acquired immune response, including in the gut and respiratory tract^36^. In previous studies, *Lactobacillus* GG has been found to cause enhanced IgA production^37^, whereas *L. Johnsonii* leads to enhanced IgA production in PP whole-organ culture supernatants^38^. DCs may be associated with the mechanism underlying the enhanced IgA production by *L. plantarum* AYA^39^. Furthermore, we and other researchers have recently demonstrated that the oral administration of recombinant *L. plantarum* may contribute to the production of secretory IgA antibodies at the mucosal surface of the respiratory tract to combat infection with pathogens such as influenza virus^25^,^40^,^41^. In this study, we extended our earlier findings and determined that NP-M1-DCpep delivered by *L. plantarum* significantly elicits B220^+^ IgA^+^ cells in the PP and ileum (data not shown). Our results indicate that NC8-pSIP409-NP-M1-DCpep induces significantly higher levels of total and antigen-specific secretory IgA in faecal matter and the BALF in mice. Cytokines such as IL-4 and TGF-β are associated with the differentiation of B cells and the induction of IgA^42^,^43^. Our data also revealed that vaccination with recombinant *L. plantarum* likely contributes to the increased numbers of FAS^+^ PNA^+^ B220^+^ B cells in the PPs and MLNs to shape GCs within the mucosa in mice. We presume that recombinant *L. plantarum* or probiotics induce T follicular helper (Tfh) cells, which contribute to T cell-dependent humoral immune responses by providing helper T cells to B cells and by contributing to GC formation and long-lived antibody responses^44^.

Earlier studies have suggested that antigen-specific T cell responses occur in most vaccinated animals and patients and could play an important role in protective immunity against infection with pathogens, including AIVs. IFN-γ producing T lymphocytes result in an enhanced specific killing function that effectively attacks AIVs, modulates chemotaxis and improves antigen presentation to elicit Th1 immune responses against AIV infection^45^. A previously published study of chickens vaccinated with MVA and in an adenovirus incorporating the NP+M1 construct has reported markedly improvedex vivo T cell IFN-γ ELISpot responses to NP and M1 peptides^46^. As determined by an IFN-γ ELISPOT assay, the oral administration of *L. plantarum* expressing NP-M1-DCpep significantly enhanced immunity in the spleen and MLNs in response to stimulation with the NP and M1 epitopes. Interestingly, our data also support recent findings that IFN-γ-secreting CD4^+^ and CD8^+^ T cells provide a protective immune response against influenza virus in humans^47^. In addition, CD4^+^ and CD8^+^ T cells efficiently responded to the NP and M1 epitopes after the final immunization. These data indicate that these specific T cell responses might be associated with the development of protective immunity after oral vaccination with recombinant *L. plantarum*.

Vaccine-induced CD8^+^ T cells might offer protection against lethal influenza virus challenge^48^, and CTLs are more important than other cell types in mediating survival in mice^49^. We previously demonstrated that the production of HA-specific CD8^+^ T cells *in vitro* also confers protection against lethal AIV (mouse-adapted H9N2) infection in mice, indicating a direct role of CD8^+^ T cells in improving mouse survival^25^. Perforin-deficient mice exhibit decreased cytotoxic activity *in vitro* or *in vivo*^50^. CD8^+^ T cells are cytolytic, and combatting cells infected with AIV or other viruses is generally considered their main role^49^. Perforin could induce this activity, resulting in the complete maturation of effector CD8^+^ T cells. The specific expression of CD107a or IFN-γ/TNF-α on CD8^+^ T cells is critical to determining the phenotype of the CD8^+^ T cells produced during our immunization schedules and associated with a protective immune response^51^. In this study, we observed that recombinant *L. plantarum* elicits the production of CD8^+^ CTLs with an activated type that may present cytotoxic activity to kill targets of the NP and M1 peptides *in vivo*. Our data are consistent with results from previous studies indicating that immunization with *Lactococcus lactis* expressing NP with a cholera toxin B subunit adjuvant in mice induces significant cellular immune responses at day 33 after the initial immunization^52^. The role of perforin in this response awaits further confirmation. Future studies should evaluate potential improvements in the survival of IFN-γ-deficient or perforin-deficient mice. In addition, a study conducted by Seder’s group suggests that vaccine-induced, multifunctional CD8^+^ cells secreting IL-2, TNF-α and IFN-γ play an important role in the protection against influenza virus infection^17^.

As shown in Fig. 7, the data displayed higher cytokine (IFN-γ^+^, TNF-α^+^ or IFN-γ^+^/TNF-α^+^) induction in NC8-pSIP409-NP-M1-DCpep-immunized mice than in other groups, such as those treated with the inactivated vaccine. The reason for this finding may be that the cells from the tissue were incubated with a mixed pool of NP and M1 peptides and an antibody against CD28, which resulted in the *L. plantarum*-expressed NP-M1-DCpep inducing more antigen-specific cytokines than the inactivated vaccine, which are based on the surface antigen-induced antibody immune response. These findings are consistent with previous research showing that CD28 signalling contributes to improving the survival of effector T cells in the lung^53^; however, the inhibition of CD28 signalling at the time of effecter T cell infiltration into the lung markedly decreases the effector cytokine secretion after influenza virus challenge^54^.

Many researchers have reported that the ratio of CD4^+^/CD8^+^ cells is indicative of the general immune system status^55^,^56^. Our data indicated that the ratio of CD4^+^/CD8^+^ cells did not markedly change in the spleens of the NC8-pSIP409-NP-M1-Ctrlpep group and the NC8-pSIP409-NP-M1-DCpep group. However, the ratios in the dLN and MLN were markedly reduced in the NC8-pSIP409-NP-M1-Dcpep group compared with the control group. No specific data have been reported regarding the roles of different AIV vaccinations on the CD4^+^/CD8^+^ ratio in dLN and MLN in recently published studies, because the cells from the tissues were cocultured with a mixed pool of NP and M1 peptides, which resulted in the *L. plantarum*-expressed NP-M1-DCpep inducing more antigen-specific CD8^+^ cells than CD4^+^ cells. These findings were consistent with the proliferation of antigen-specific CD8 T cells in dLNs after intranasal influenza HA/M1-VLP administration^57^.

As shown in Fig. 9, the injection of CD8^+^ T cells derived from BALB/c mice pre-treated with recombinant *L. plantarum* into NOD-SCID mice improved their weight loss and survival after a subsequent challenge with mouse-adapted H9N2 AIV. The transfer experiments with NOD-SCID mice provide additional evidence that CD8^+^ T cells but not CD4^+^ T cells play an important role in combatting influenza viruses. CD8^+^ T cell responses can provide strong protection against influenza virus infections^48^. Furthermore, specific CD8^+^ T cells result in strong pathogen-killing effects, and thus, the use of these T cells has been studied extensively^58^,^59^. However, whether CD4^+^ T cells provide protective responses to influenza virus in mice vaccinated with recombinant *L. plantarum* via other mechanisms awaits further study.

The oral administration of lactobacillus has been shown to offered effective protection against influenza virus infection in a mouse model. Mice were orally administered LG2055 or heat-killed *Lactobacillus pentosus* b240 once a day for 21 days and were subsequently i.n. inoculated with the influenza virus, and some of the observed protection may have resulted from non-antigen-specific immune responses to the lactobacillus^60^,^61^. In our experiment, however, the mice were immunized orally with recombinant *L. plantarum* and vaccine vectors using a primer-booster regime. In addition, the mice immunized with NC8-pSIP409-NP-M1-DCpep started to recover eight days after challenge, and all of these mice survived (Fig. 10a,b), indicating a protective effect against homologous AIV challenge. However, the immunized mice challenged with 10 × LD_50_ heterologous virus did not exhibit effective protection. Recombinant *L. plantarum* may not protect mice against H1N1 infection because the epitopes of the NPs or M1 from the H9N2 and HIN1 subtypes of AIV differ. Therefore, although the recombinant *L. plantarum* provides only limited protection against infection with a lethal dose of a heterologous virus, it can provide complete protection against infection with a sub-lethal dose of a heterologous virus. In future studies, we will examine more conservative vaccine epitopes to develop more effective vaccines.

Different vaccines were found to offer different immuno-protective effects. Inactivated vaccines targeting AIV surface proteins, such as haemagglutinin, provided complete protection against homologous AIV (Fig. 10c), but conferred only partial protection against heterologous viral strains (Fig. 10e), to which a humoral immune response is thought to provide the major protective effect^62^. A more effective strategy aiming to stimulate T cell responses has been confirmed to provide protection against heterologous influenza virus strains in both mice and humans^63^,^64^. In this study, oral immunization with *L. plantarum* expressing NP-M1-Dcpep provided effective protection against heterologous influenza virus infection (Fig. 10e). In addition, *L. plantarum*-based vaccines have other advantages, including the protection of the integrity of the lungs, possibly because Tregs markedly improve the lungs of mice that administered *L. rhamnosus*^65^. Many studies have implicated Ag-specific Tregs in primary infections. In addition, antigen-specific memory regulatory CD4^+^Foxp3^+^ T cells contribute to immune regulation during memory responses to a previously encountered influenza virus, which play important regulatory roles in the cellular immune responses to viral infections and are thought to prevent pathological changes resulting from excessive immune responses^66^.

In conclusion, our results demonstrate that the oral administration of an NP-M1-DCpep-expressing bacterial vector can provide protection against infection with a lethal dose of mouse-adapted H9N2 AIV or a sub-lethal dose of a heterologous virus by increasing the mucosal and adaptive immune responses. Oral vaccination resulted in significant increases in DC activation, specific sIgA antibody production, CD8 T cell induction and cross-protection against viral challenge *in vivo*.

## Methods

### Animals and ethics statement

Six- to eight-week-old female C57BL/6, BALB/c and NOD.CB17-Prkdc^scid^/NcrCrl mice were obtained from Beijing Vital River Laboratory Animal Technology Co., Ltd., China, and bred in a pathogen-free animal facility at Jilin Agriculture University. All animal experiments were managed in accordance with the approval of the Animal Care and Use Committee of Jilin Agriculture University (JLAU08201409 for mice), and the animal facility was completely accredited by the National Association of Laboratory Animal Care.

### Virus and peptide

H9N2 AIV (A/duck/Xuzhou/07/2003) and H1N1 influenza (A/PR/8/34) viruses were adapted to grow in mice as previously described^25^. The peptides NP_366–374_ (ASNENMEAM) of NP and M1_26–37_ (RAVKLYKKLKRE) of M1 from H9N2 AIV (A/duck/Xuzhou/07/2003) were synthesized using a peptide synthesizer (Shanghai ZiYu Biotech Co., Ltd.).

### Generation of recombinant NC8-pSIP409-NP-M1-DCpep

To fuse M1 of H9N2 AIV A/duck/Xuzhou/07/2003 to 12 amino acids (DC peptide, FYPSYHSTPQRP) derived from a phage display library or the control peptide (EPIHPETTFTNN) at the C terminus as previously described^67^, two shuttle plasmids (pSIP409-M1-DCpep and pSIP409-M1-Ctrlpep) were generated. Subsequently, the NP gene from A/duck/Xuzhou/07/2003 was amplified by PCR (primers: 5-CTGGTCGCTTCCGTGCTACCTAGAACTAAATGCATG-3, 5-AGATCCCGAGCCACCTCCTCCGGACCCACCCCCGCCTGATCCCAAAACTCTTGCCTTATG-3) from the pMD18T-NP plasmid, which was constructed by our laboratory group. The reverse primer amplifies a linker containing 13 codons for glycine and serine residues (GSSGGGSSGGSSS). The gene encoding the NP fusion was subsequently subcloned into the *Nco*I and *Xho*I sites of pSIP409-M1-DCpep (or pSIP409-M1-Ctrlpep) to generate pSIP409-NP-M1-DCpep (or pSIP409-NP-M1-Ctrlpep). The recombinant plasmids pSIP409-NP-M1-DCpep and pSIP409-NP-M1-Ctrlpep were then electroporated into *L. plantarum* NC8. ERM-resistant positive clones were selected and verified by DNA sequencing.

### Western blotting

To detect NP and M1 antigen expression by *L. plantarum* NC8, NC8-pSIP409-NP-M1-DCpep, NC8-pSIP409-NP-M1-Ctrlpep and NC8-pSIP409 were cultured and induced with 10 μg/ml erythromycin and 50 ng/ml Sakacin P (SppIP) as previously described. After culturing at 37 °C for 8 h, the recombinant *L. plantarum* was harvested and disrupted on ice by sonication. After separation by SDS-PAGE (10% acrylamide), the bacterial proteins were transferred to nitrocellulose membranes and incubated with a monoclonal mouse anti-NP or M1 antibody and then with a secondary horseradish peroxidase (HRP)-conjugated goat anti-mouse antibody (Cell Signaling Technology, CST). After washing, the blots were visualized by enhanced chemiluminescence (ECL, Thermo Scientific) on an Amersham Imager (General Electric Company).

### Immunization

Female C57BL/6 or BALB/c mice were segregated into five groups. Four of the groups of mice received PBS, NC8-pSIP409, NC8-pSIP409-NP-M1-Ctrlpep or NC8-pSIP409-NP-M1-DCpep by gavage. The remaining group of mice was intramuscularly administered H9N2 AIV inactivated vaccine (Weike Biotech, China) (50 μl/mouse). In brief, recombinant *L. plantarum* was cultured at 30 °C in MRS broth containing 10 μg/ml erythromycin and 50 ng/ml SppIP for 12 h under anaerobic conditions. The freshly cultured recombinant *L. plantarum* was then washed with phosphate-buffered saline (PBS) and resuspended in 250 μl of PBS for the administration of 1.0 × 10^9^ cfu per mouse. Recombinant *L. plantarum* was orally administered by gavage three times on days 1, 2 and 3, and the four groups of mice received three booster vaccinations four weeks later on days 28, 29 and 30.

### Mouse DC culture and flow cytometry

Mouse bone marrow-derived DCs were induced and purified according to a published protocol^22^,^68^. DC activation was confirmed by co-culturing mouse DCs with NC8-pSIP409, NC8-pSIP409-NP-M1-Ctrlpep and NC8-pSIP409-NP-M1-DCpep at a ratio of 1:10 for 12 h at 37 °C. DCs from bone marrow and other tissues were labelled with an allophycocyanin (APC)-conjugated antibody for CD11c (clone HL3) together with one of the following: CD40 (clone 3/23), CD80 (clone 16–10A1), CD86 (clone GL1), or MHC-II (clone 3/23). B220^+^ CD95^+^ PNA^+^ cells were detected as previously described using a BD Accuri C6 flow cytometer (BD Biosciences)^25^. The data were analysed with FlowJo 7.6.2 software.

### IFN-γ ELISPOT assay.

The IFN-γ ELISPOT assay was employed as previously described^25^. Murine MLN cells and splenocytes were seeded on IFN-γ-coated ELISPOT plates (Mabtech) and incubated with NP and M1 peptides at a final concentration of 5 μg/ml for 20 h. The control wells did not contain peptide. After incubation with the detection antibodies (R46A2), streptavidin-horseradish peroxidase (1 μg/ml) and substrate solution were added. The IFN-γ spot-forming cells (SFC) were detected using an automated spot detection device (AID).

### Intracellular cytokine staining (ICS)

ICS was performed as previously published protocol^31^. Leukocytes (1.0 × 10^6^) from the spleen, MLN or lung were incubated with a mixed pool of 20 μg/ml NP and M1 peptides and 2 μg/ml antibody against CD28 (BD Pharmingen) or media only (as a control) for 2 h at 37 °C. Golgi-Stop and Golgi-Plug (BD Pharmingen) (0.2 μl of Golgi-Stop and 0.2 μl of Golgi-Plus per 1.0 × 10^6^ leukocytes) were added 4 h before ICS. The cells were then blocked at 4 °C with 2.4G2 mAb and incubated with fixable viability stain 450 (BD Horizon™, Cat. No. 562247). Following surface staining with PerCP-Cy5.5-CD3e (clone 145-2C11), APC-Cy7-CD8 (clone 53-6.7), and PE-Cy7-CD4 (clone OX-35) (BD Pharmingen), the cells were fixed with the BD Cytofix/Cytoperm kit following the manufacturer’s protocol and labelled intracellularly using PE-conjugated anti-IL-2 (clone JES6–5H4), APC-conjugated anti-IFN-γ (clone XMG1.2), FITC-conjugated anti-TNF-α (clone MP6-XT22) or isotype control PE-, APC-, and FITC-conjugated antibodies (BD Pharmingen). Finally, the samples were analysed with a BD FACS Aria™ II flow cytometer, and the data were analysed using FlowJo 7.6.2 software.

### *In vivo* cytotoxic assay

The specific cytotoxic T lymphocyte (CTL) assay was conducted according to a published protocol^51^. In brief, splenocytes isolated from naive BALB/c mice were divided into two groups and stained with carboxy fluorescein diacetate succinimidyl ester (CFSE, Invitrogen) at a final concentration of 0.5 μM (CFSE^Low^) or 5 μM (CFSE^High^) to obtain the targets. The CFSE^High^ cells were then incubated with 10 μg/ml NP and M1 peptide for 1 h at 37 °C, whereas the CFSE^Low^ cells were not incubated with peptide. The CFSE^High^ cells were washed and mixed with the CFSE^Low^ cells in a 1:1 ratio (1.0 × 10^7^ total cells) and injected into mice via the caudal vein. After 18 h, the CFSE^High^- and CFSE^Low^- stained cells in the splenocytes of all groups of mice were collected and detected using a BD Accuri C6 (BD Biosciences). The level of specific killing was evaluated based on the following formula: % specific killing = [1 − (% CFSE^High^ vaccinated/% CFSE^Low^ vaccinated)/(% CFSE^High^ naive/% CFSE^Low^ naive)]  × 100.

### T cell proliferation assay

To assess the proliferation of the primed CD4^+^ and CD8^+^ T cells, murine splenocytes and cells from the dLNs and MLNs were stained with CFSE (Invitrogen) for 10 min at 37 °C and incubated with anti-CD28 and either NP and M1 peptide or anti-CD3 as a positive control in 96-well plates. After 72 h of incubation, the cells were labelled with APC-conjugated anti-CD3 and PE-conjugated anti-CD4 or anti-CD8 as described above (BD Biosciences). Finally, the samples were analysed using a BD FACSAria™ II flow cytometer, and the data were analysed using FlowJo software.

### CD4^+^ or CD8^+^ T cell isolation and adoptive transfer

MLNs and spleens from BALB/c mice vaccinated with PBS, NC8-pSIP409, NC8-pSIP409-NP-M1-Ctrlpep or NC8-pSIP409-NP-M1-DCpep were aseptically collected into RPMI 1640 (GIBCO) with 8% FCS (GIBCO). To obtain MLN cells and splenocytes, the organs were gently crushed using a 70-μM porefilter (BD Falcon), and the red blood cells (RBCs) were then removed using ACK lysis buffer (BD Pharm Lyse^TM^). Following positive selection via a magnetic cell-sorting column (MACS) according to the manufacturer’s methods, CD4^+^ or CD8^+^ T cells were incubated with microbead-conjugated anti-CD4 or anti-CD8 MAb (Miltenyi). After analysing the purity of the CD4^+^ and CD8^+^ T cells, purified CD4^+^ T cells alone, purified CD8^+^ T cells alone or equal amounts of CD4^+^ T cells plus CD8^+^ T cells (2 × 10^6^ cells/mouse) were a doptively transferred into SPF NOD/SCID mice (age-matched recipients) via tail vein injection.

### Influenza virus challenge

Four weeks after the booster vaccination, all animals were anesthetized intraperitoneally (i.p.) with 15 mg/kg mebubarbital (Avertin, Sigma, USA). Groups of C57BL/6 mice were intranasally (i.n.) inoculated with 10 × LD_50_ of mouse-adapted H9N2 AIV (A/duck/Xuzhou/07/2003) or H1N1 influenza virus (A/PR/8/34). After adoptive transfer for 24 h, all groups of recipient NOD/SCID mice were i.n. inoculated with 0.5 × LD_50_ of mouse-adapted H9N2 AIVs under anaesthesia. The mice challenged with the virus were observed daily for two to four weeks, and changes in their weight and mortality were noted. To determine the TCID_50_ of the virus in mouse lung tissue, a sample was plated in Madin-Darby canine kidney (MDCK) cells and incubated for at least three days. The TCID_50_ values were determined using a published protocol^69^. A weight loss of 20% was considered a humane endpoint, and mice satisfying this standard were euthanized with an overdose of mebubarbital as previously described.

### Histopathological detection

To evaluate pathological damage of the lung after mouse-adapted H9N2 or H1N1 virus challenge, pulmonary samples were removed from the mice, fixed in 4% paraformaldehyde and embedded in paraffin. Sections with a thickness of 8 μm were stained with haematoxylin and eosin (H&E) and scored by blinded pathologists (three independent readers). Briefly, alveolitis and peribronchiolar inflammation was blind scored on ascale of 0, 1, 2, 3, 4 or 5 corresponding to none, very mild, mild, moderate, marked, or severe inflammation, respectively^70^.

### Statistical analysis

All values represent the means ± standard error of the mean (SEM) of at least three independent experiments. Two-tailed *t*-tests and ANOVA were employed for the statistical analyses, which were performed using GraphPad Prism 5 software. Mortality was analysed using the Kaplan-Meier method with the log-rank test. *P* < 0.05 was considered significant.

## Additional Information

**How to cite this article**: Yang, W.-T. *et al*. Cross-protective efficacy of dendritic cells targeting conserved influenza virus antigen expressed by *Lactobacillus plantarum. Sci. Rep.* **6**, 39665; doi: 10.1038/srep39665 (2016).

**Publisher's note:** Springer Nature remains neutral with regard to jurisdictional claims in published maps and institutional affiliations.

## Supplementary Material

Supplementary Information
