# Supplementary material for: Cross-protective efficacy of dendritic cells targeting conserved influenza virus antigen expressed by Lactobacillus plantarum
Source: Sci Rep. 2016 Dec 22;6:39665. doi: 10.1038/srep39665 (PMC5177883; doi:10.1038/srep39665)
Supplement: Supplementary Information [file srep39665-s1.pdf]

Supplementary Information

Cross-protective efficacy of dendritic cells targeting conserved influenza virus antigen expressed by *Lactobacillus plantarum*

Wen-Tao Yang, Shao-Hua Shi, Gui-Lian Yang, Yan-Long Jiang, Liang Zhao, Yu Li, Chun-Feng Wang*

College of Animal Science and Technology, Jilin Provincial Engineering Research Center of Animal probiotics, Engineering Research Center of Chinese Ministry of Education for Edible and Medicinal Fungi, Jilin Agricultural University, Changchun, 130118, China

*Corresponding authors: Chun-Feng Wang, College of Animal Science and Technology, Jilin Provincial Engineering Research Center of Animal probiotics, Jilin Agricultural University, 2888 Xincheng Street, Changchun 130118, China. E-mail: wangchunfeng@jlau.edu.cn, Tel./fax: +86 43184533425;

Supplementary Methods

Ab assays

IgA Abs against NP and M1 were detected in faecal and BALF samples obtained from the mice on day 14 after the final vaccination. Specific anti-NP and anti-M1 IgA Abs were determined by ELISA as previously described[1]. In brief, ELISA plates were coated with 2 μg of purified NP and M1 antigen per well. The appropriate dilutions of samples from each individual mouse were applied to the plates, serially diluted two-fold in PBS/Tween (PBS containing 0.05% Tween 20), and then incubated for 1 h at 37°C. Subsequently, the plates were washed and incubated with horseradish peroxidase-conjugated goat antibodies directed against IgA. After incubation with mouse IgA conjugates for 1 h at 37°C, the plates were washed twice with PBS/Tween and once with PBS. Antibodies were detected using substrate buffer (50 mM phosphate buffer pH 5.5 containing 0.02% OPD and 0.006% perhydrol). Plates were developed in the dark for 30 min at room temperature, after which the reaction was stopped by the addition of 50 μl 2 M H_2SO_4 per well, and absorbances were read at 492 nm (A492) using a SPECTRA I ELISA reader. The titers were defined as end-point titers, and the absorbance of the highest dilution was two-fold greater than the background value of the faecal and BALF samples.

Immunofluorescence microscopy

Sections (7 μm) of mouse ileum were cut and fixed with 4% formaldehyde for 15 min at room temperature. The sections were then washed and blocked with blocking buffer (PBS containing 1% BSA and 0.3% tritonX-100) for 60 min. The tissue

sections were incubated with rabbit anti-mouse CD79a (BD Pharmingen) and rat anti-mouse B220 (BD Pharmingen) overnight at 4°C. They were then rinsed three times in PBS for 5 min each and incubated with Cy3-conjugated goat anti-rabbit IgG (Cell Signaling Technology) or FITC-conjugated goat anti-Rat IgG (Abcam). Sections were analysed by confocal microscopy using a Zeiss LSM710 microscope.

References

1. de Haan, L. et al. Nasal or intramuscular immunization of mice with influenza subunit antigen and the B subunit of *Escherichia coli* heat-labile toxin induces IgA-or IgG-mediated protective mucosal immunity. *Vaccine* **19**, 2898-2907 (2001).

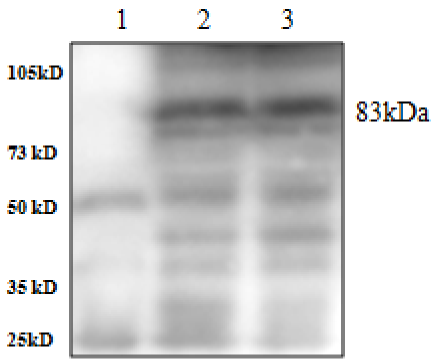

Supplementary Figure S1. Western blotting of the synthesized proteins. All cultures were induced with SppIP for 8h and then subjected to an immunological assay using an anti-M1 prime antibody. Lane 1: negative control, NC8 (pSIP-409); lane 2: NC8 (pSIP409-NP-M1-Ctrlpep); and lane 3: NC8 (pSIP409-NP-M1-DCpep).

a

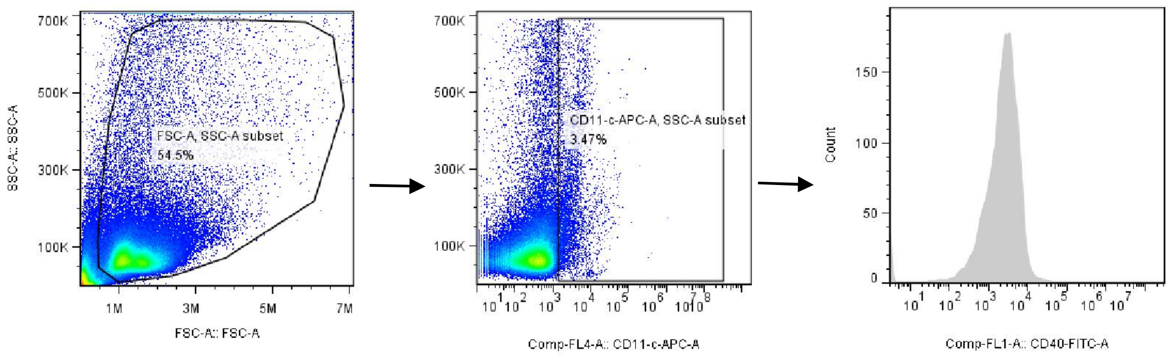

FSC-A, SSC-A subset
54.5%
SSC-A:: SSC-A
FSC-A:: FSC-A
CD11-c-APC-A, SSC-A subset
3.47%
Comp-FL4-A:: CD11-c-APC-A
Count
Comp-FL1-A:: CD40-FITC-A

b

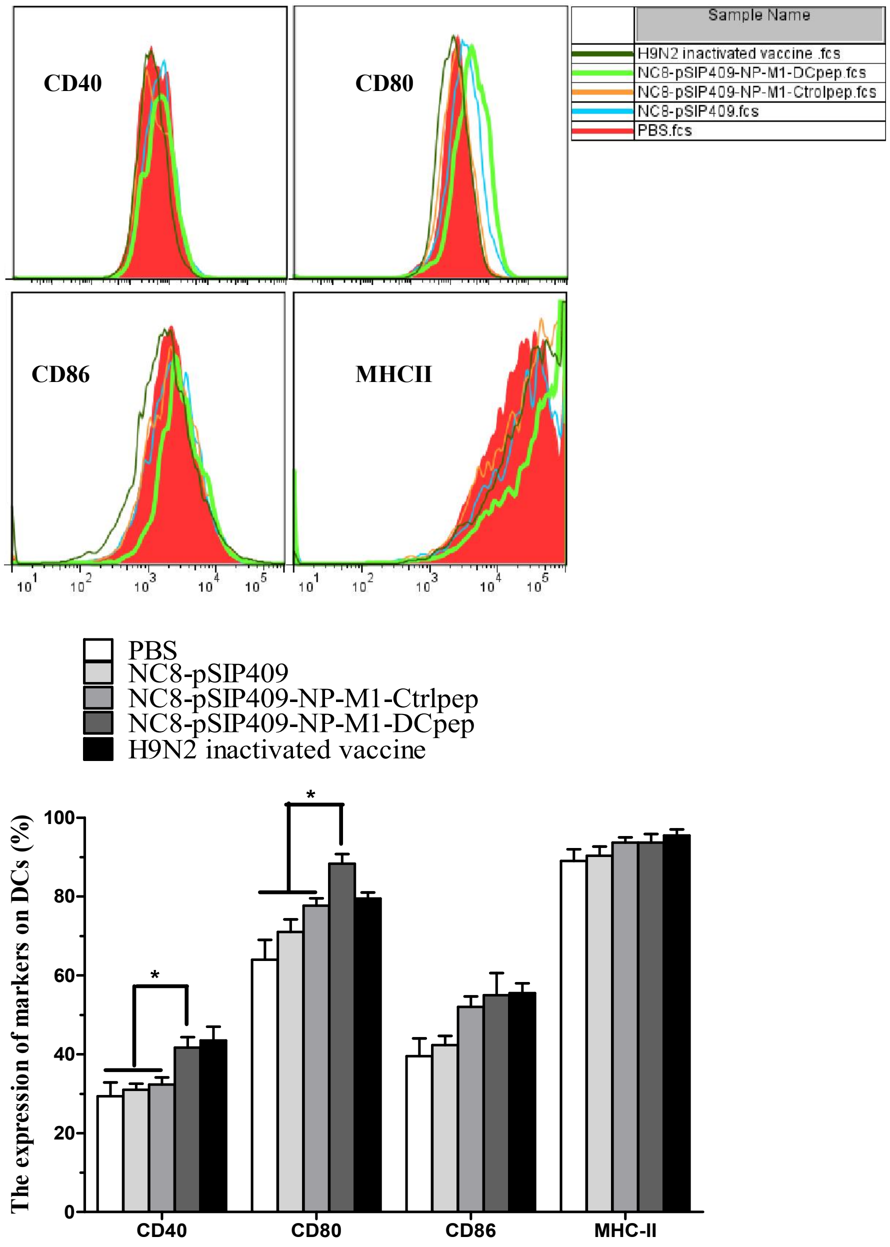

CD40
CD80
CD86
MHCII
Sample Name
H9N2 inactivated vaccine .fcs
NC8-pSIP409-NP-M1-DCpep.fcs
NC8-pSIP409-NP-M1-Ctrolpep.fcs
NC8-pSIP409.fcs
PBS.fcs
PBS
NC8-pSIP409
NC8-pSIP409-NP-M1-Ctrlpep
NC8-pSIP409-NP-M1-DCpep
H9N2 inactivated vaccine
The expression of markers on DCs (%)
CD40
CD80
CD86
MHC-II

c

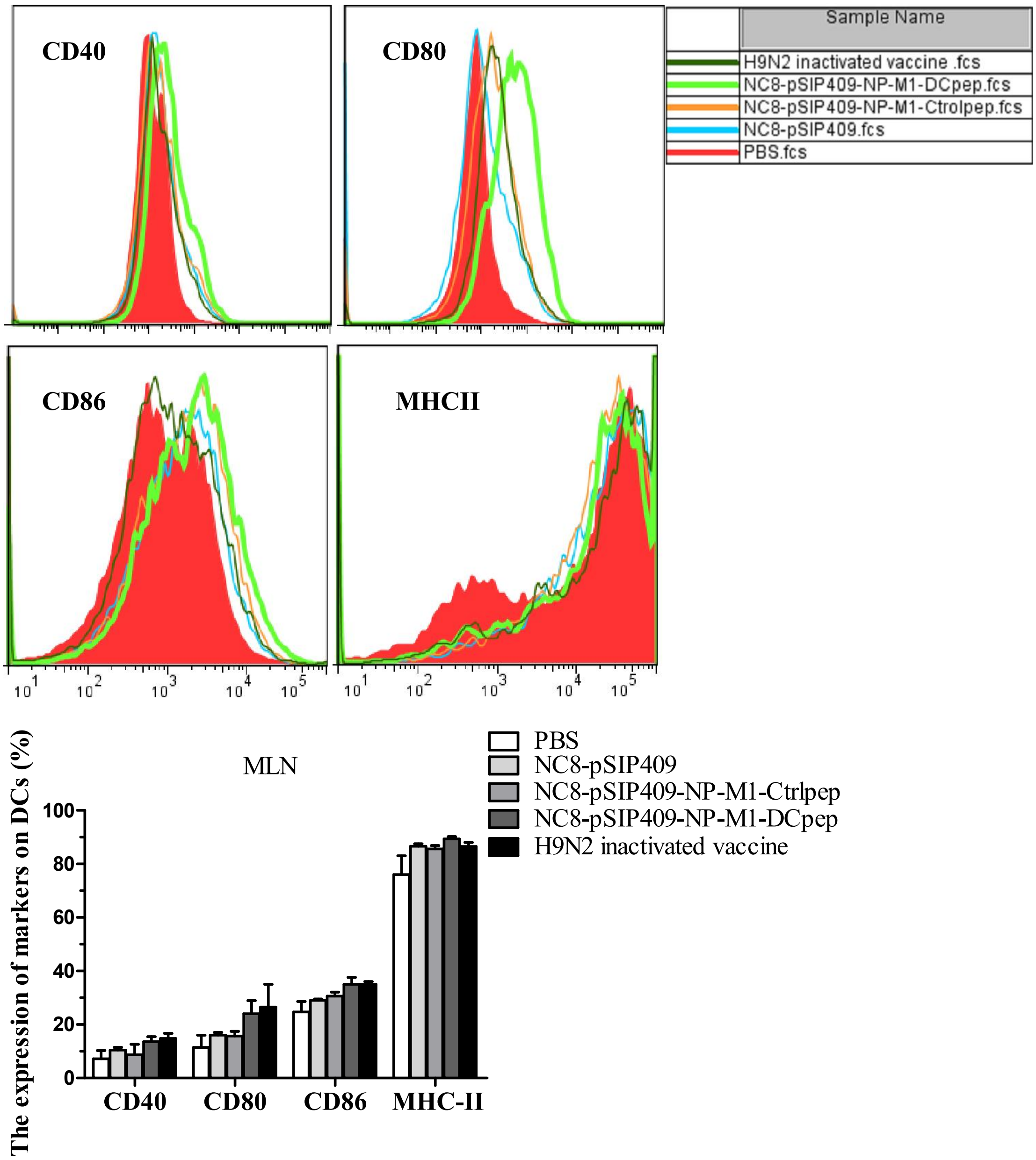

Supplementary Figure S2. Upregulated expression of CD80, CD86, CD40 and MHC-II on the surface of DCs. (a) DCs gating strategy. (b) and (c) DCs were also isolated from PP and MLN of mice immunized with recombinant *L. plantarum* or H9N2 inactivated vaccine for 24 h, and the expressions of CD80, CD86, CD40 and MHC-II were determined. Results are presented as the means ± S.E.M of triplicate tests (n=8-10 mice each group), and are analyzed using one way Anova, assuming Gaussian distribution followed by the Dunnett Post-test, and are expressed relative to the PBS, NC8-pSIP409 and NC8-pSIP409-NP-M1-Ctrlpep (*P<0.05). Data shown represent one of three experiments with equivalent results.

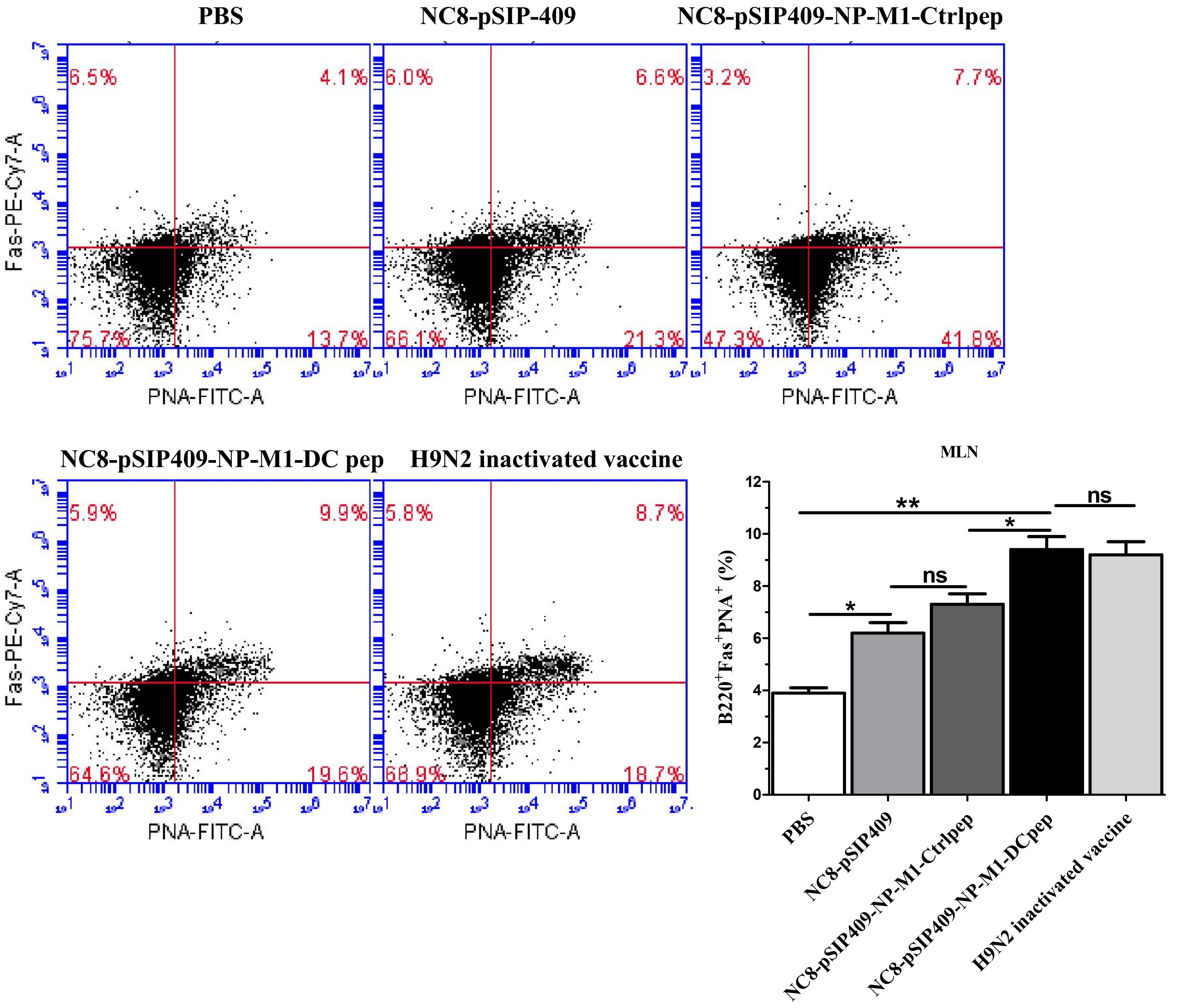
PBS
NC8-pSIP-409
NC8-pSIP409-NP-M1-Ctrlpep
6.5%
4.1%
75.7%
13.7%
6.0%
6.6%
66.1%
21.3%
3.2%
7.7%
47.3%
41.8%
Fas-PE-Cy7-A
PNA-FITC-A
NC8-pSIP409-NP-M1-DC pep
H9N2 inactivated vaccine
5.9%
9.9%
64.6%
19.6%
5.8%
8.7%
66.9%
18.7%
MLN
B220+Fas+PNA+ (%)
**
ns
*
ns
*
PBS
NC8-pSIP409
NC8-pSIP409-NP-M1-Ctrlpep
NC8-pSIP409-NP-M1-DCpep
H9N2 inactivated vaccine

Supplementary Figure S3. Determination of activated B cells in the growth center (GC) of MLNs. The GC B cells among B220$^+$ B cells (PNA$^+$ FAS$^+$) in the MLNs were determined by flow cytometer at day 5 after booster immunization. Results are presented as the means ± S.E.M of triplicate tests (n = 5 mice each group) and are analyzed using one way Anova, assuming Gaussian distribution followed by the Dunnett Post-test, and are expressed relative to the PBS, NC8-pSIP409 and NC8-pSIP409-NP-M1-Ctrlpep (* P<0.05, ** P<0.01).

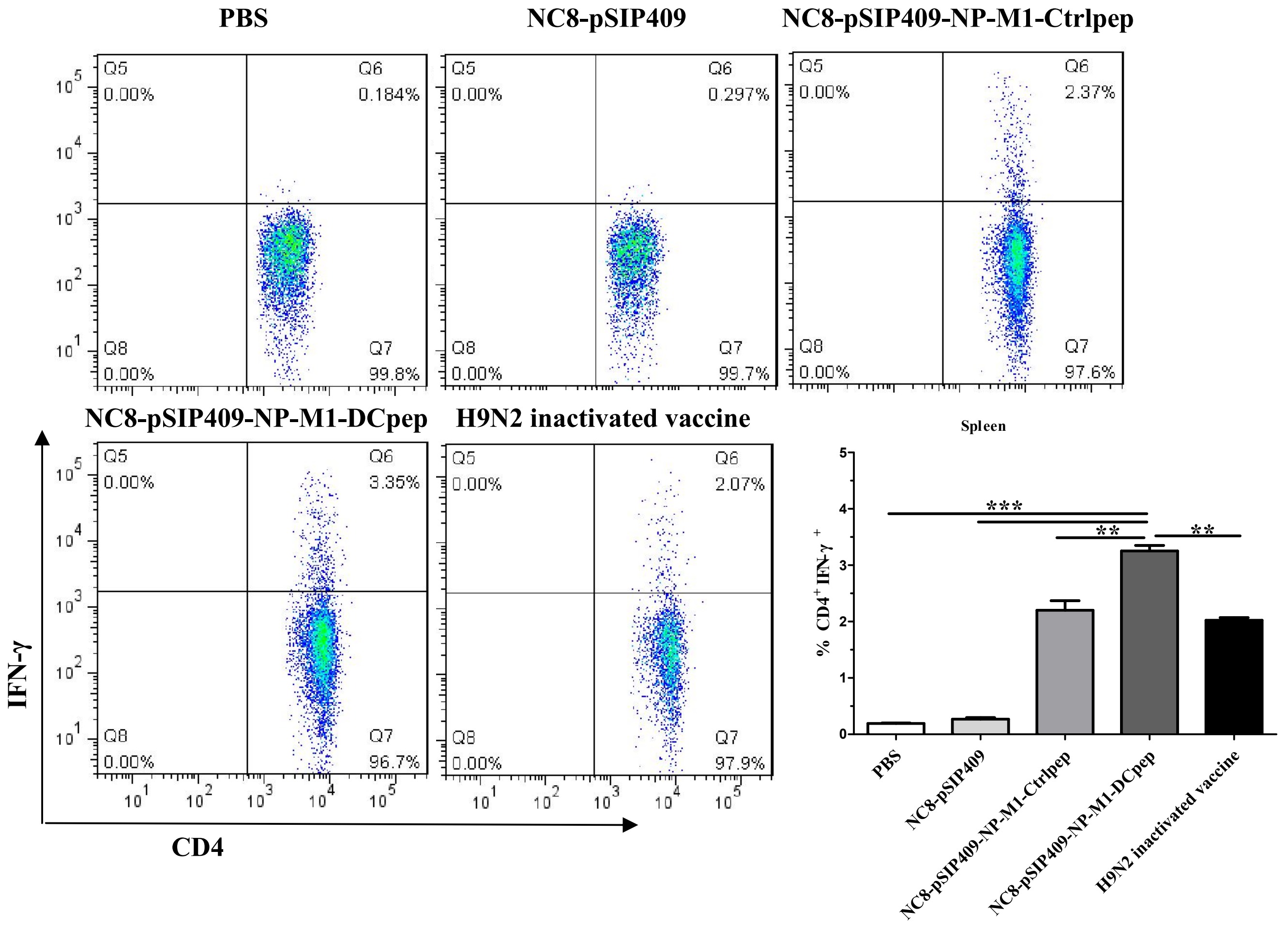
PBS
NC8-pSIP409
NC8-pSIP409-NP-M1-Ctrlpep
NC8-pSIP409-NP-M1-DCpep
H9N2 inactivated vaccine
Q5 0.00%
Q6 0.184%
Q8 0.00%
Q7 99.8%
Q5 0.00%
Q6 0.297%
Q8 0.00%
Q7 99.7%
Q5 0.00%
Q6 2.37%
Q8 0.00%
Q7 97.6%
Q5 0.00%
Q6 3.35%
Q8 0.00%
Q7 96.7%
Q5 0.00%
Q6 2.07%
Q8 0.00%
Q7 97.9%
IFN-γ
CD4
Spleen
% CD4+ IFN-γ+
***
**
**

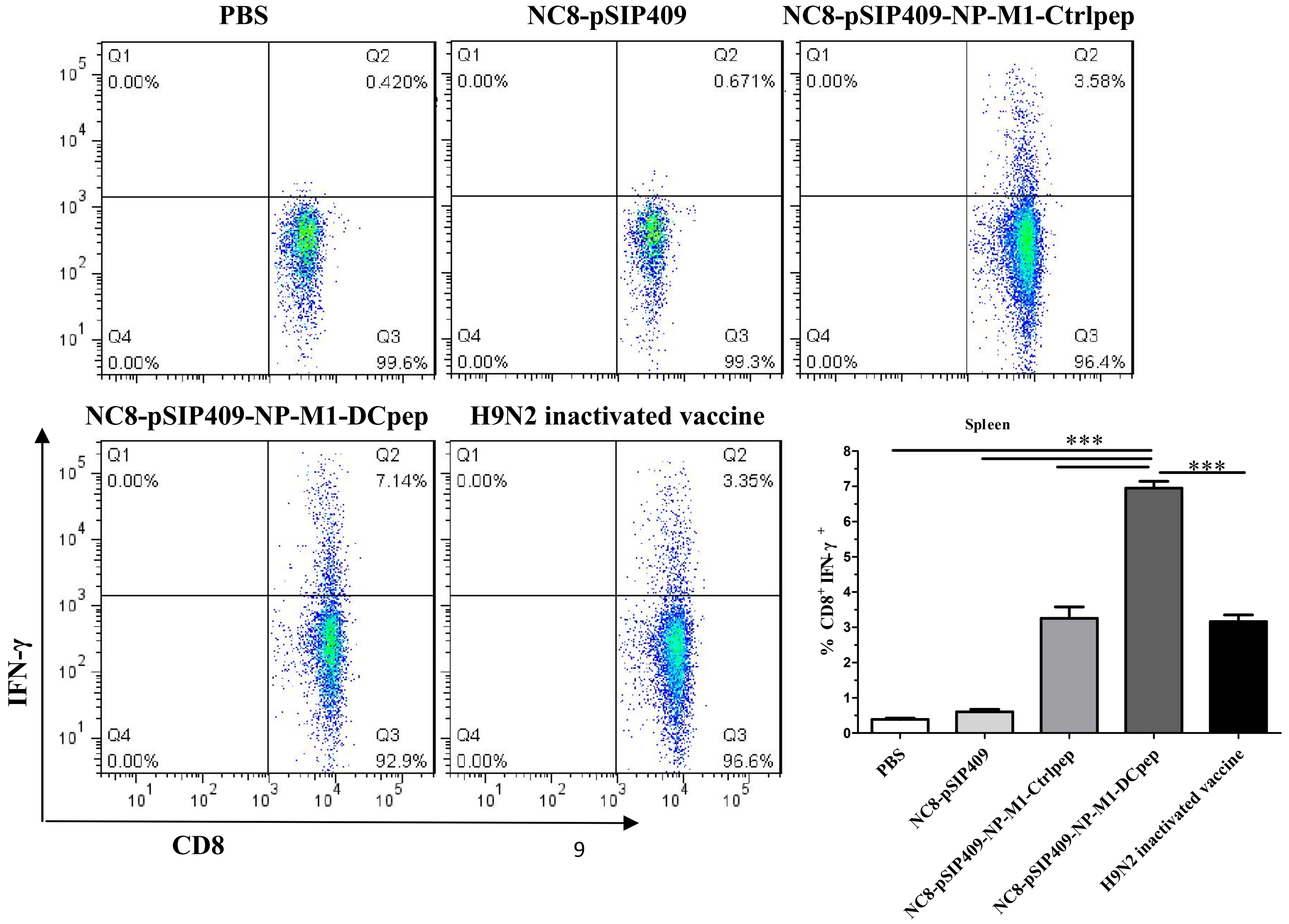
PBS
NC8-pSIP409
NC8-pSIP409-NP-M1-Ctrlpep
NC8-pSIP409-NP-M1-DCpep
H9N2 inactivated vaccine
Q1 0.00%
Q2 0.420%
Q4 0.00%
Q3 99.6%
Q1 0.00%
Q2 0.671%
Q4 0.00%
Q3 99.3%
Q1 0.00%
Q2 3.58%
Q4 0.00%
Q3 96.4%
Q1 0.00%
Q2 7.14%
Q4 0.00%
Q3 92.9%
Q1 0.00%
Q2 3.35%
Q4 0.00%
Q3 96.6%
IFN-γ
CD8
Spleen
% CD8+ IFN-γ+
***
***

Supplementary Figure S4. Oral vaccination of NC8-pSIP409-NP-M1-DCpep elicits enhanced IFN-γ-producing CD4$^+$ T cells and CD8$^+$ T cells in the spleen. The frequency of antigen-specific IFN-γ-producing CD4$^+$ T cells and CD8$^+$ T cells in the MLN was detected by flow cytometry. The data are the mean values ± SEM (n =5), and are analyzed using one way Anova, assuming Gaussian distribution followed by the Dunnett Post-test, and are expressed relative to the PBS, NC8-pSIP409, NC8-pSIP409-NP-M1-Ctrlpep and H9N2 (*** P<0.0001).

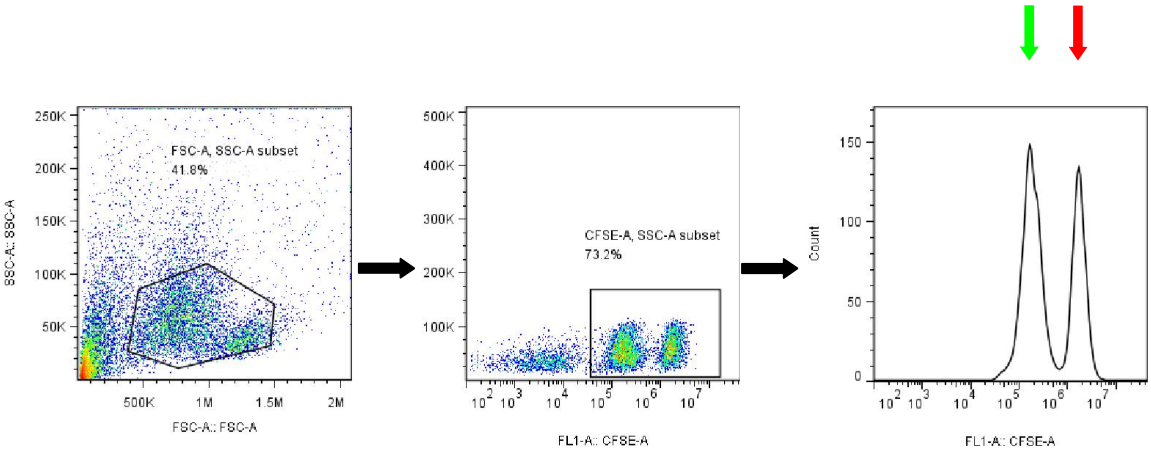

Supplementary Figure S5. The gating strategy for cytotoxic assay. The splenocytes from naive mice stained with CFSE were injected in vaccinated mice following coat with the NP and M1 peptide as. The in vivo cell-mediated cytotoxicity was characterized at 18 h. The green arrow represents the CFSE^{low} cells that were not incubated with peptide, and the red arrow shows NP and M1-pulsed, CFSE^{High} target cells.

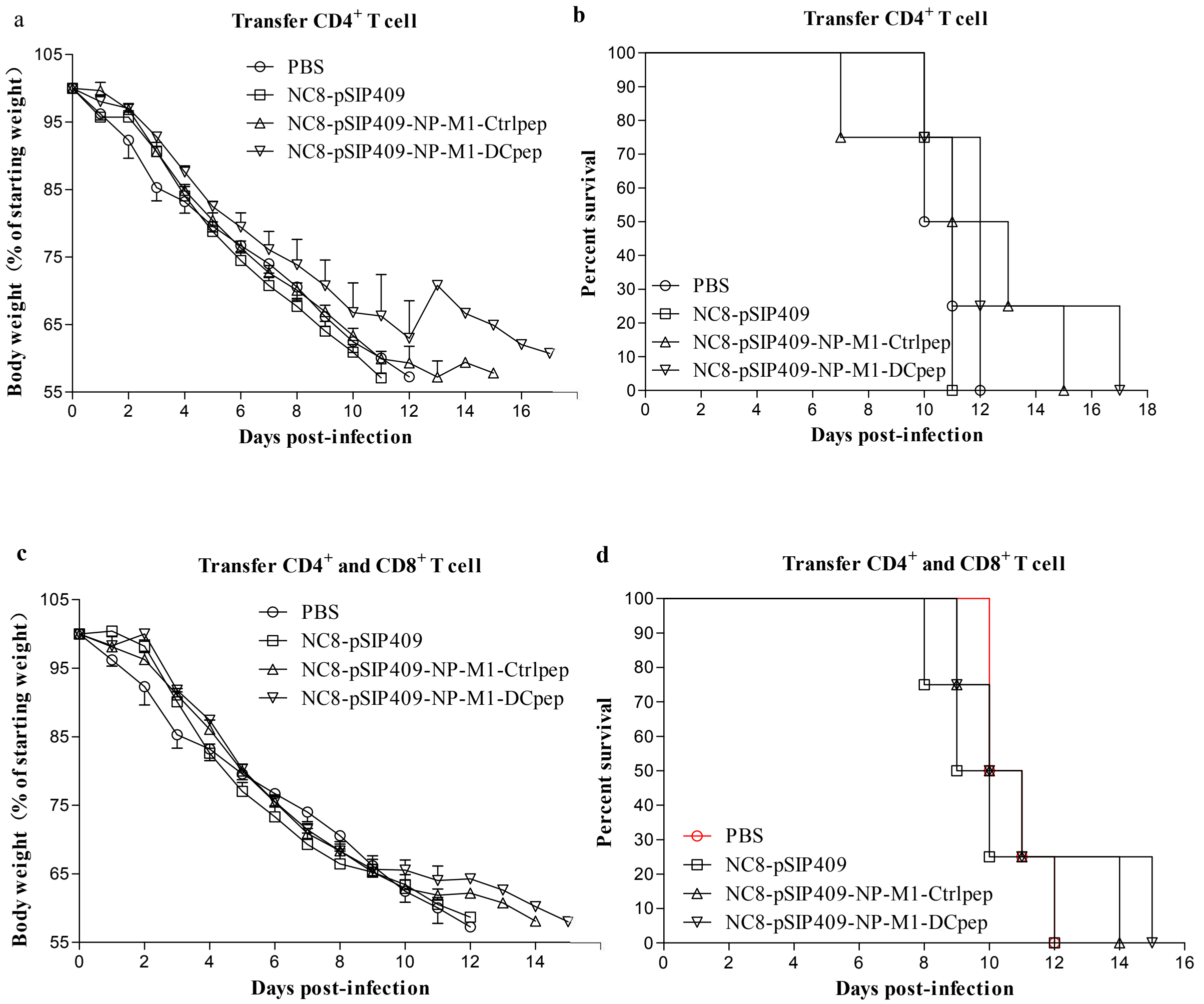

Supplementary Figure S6. Protective effect of transferred NC8-pSIP409NP-DCpep-primed CD4$^+$ T cells or CD4$^+$ plus CD8$^+$ T cells in NOD/SCID mice. CD4$^+$ and CD8$^+$ T cells were isolated from spleen and MLN of mice immunized with strains as indicated 14 days after booster vaccination, and then adoptively transferred to NOD-SCID mice by i.v. (tail vein) injection. (a) and (c) The weight losses of mice infected with mH9N2 virus were measured daily until day 18. (b) and (d) Survival percents of NOD/SCID mice was monitored for four weeks after infection. The results are presented as the mean±SEM of triplicate tests (n=5 mice per group).

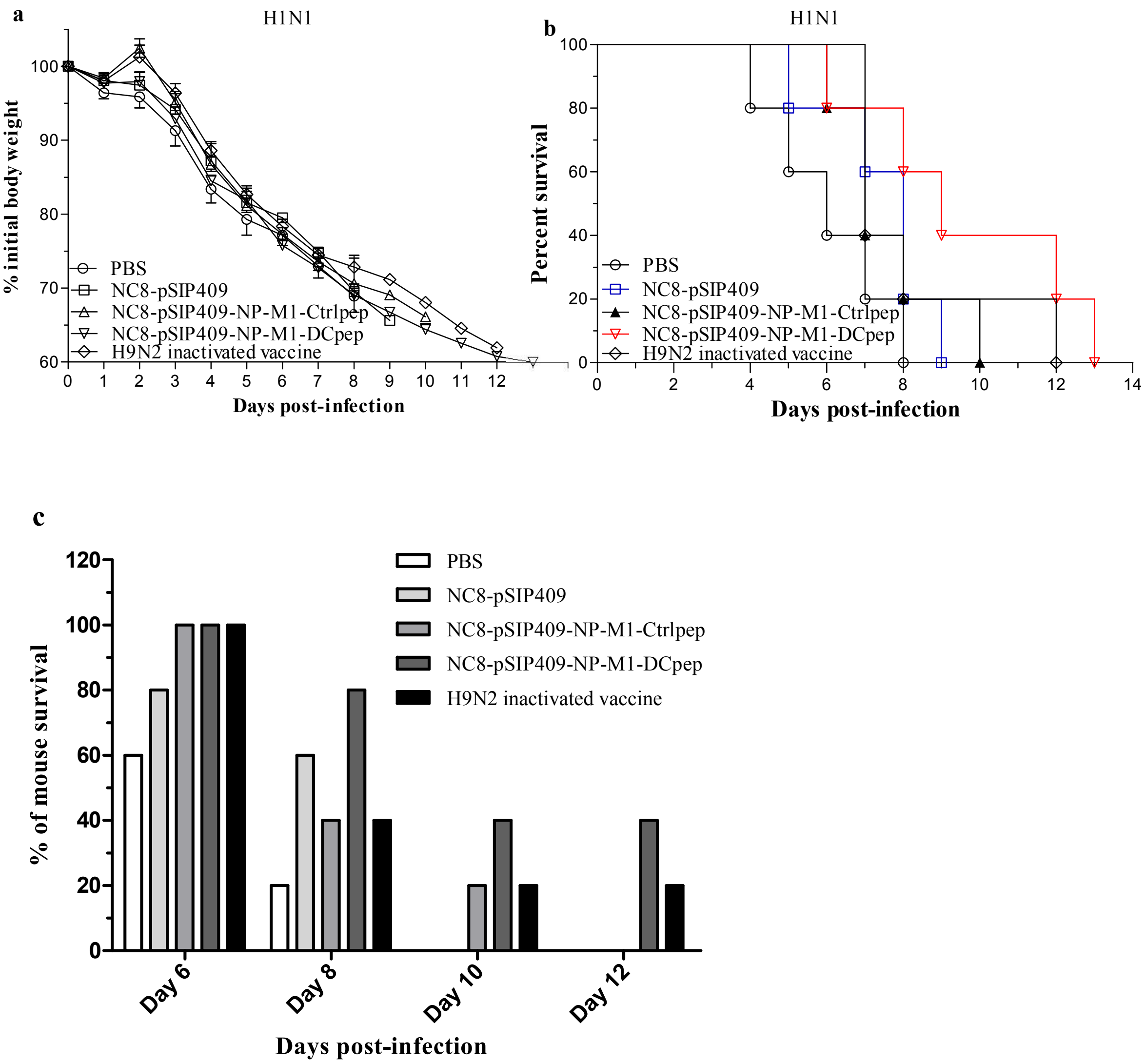

Supplementary Figure S7. Oral vaccination of recombinant *L. plantarum* protects mice from heterologous influenza virus infections. Groups of C57BL/6 mice were immunized with recombinant *L. plantarum*. Four weeks after vaccination, mice were infected with A/PR/8/34(H1N1) (10×LD_{50}) and the weight loss (a) and survival percents (b and c) were recorded for 14 days (n=10 mice per group).
